# A Review of the Nutritional Composition, Storage Challenges, Processing Technology and Widespread Use of Bamboo Shoots

**DOI:** 10.3390/foods13223539

**Published:** 2024-11-06

**Authors:** Ting Ma, Wenfeng Mo, Beibei Lv, Wenxuan Wang, Hailin He, Cuiwen Jian, Xiaoling Liu, Shubo Li, Yuan Guo

**Affiliations:** 1 College of Light Industry and Food Engineering, Guangxi University, Nanning 530004, China; mating@st.gxu.edu.cn (T.M.); mowenfeng1030@163.com (W.M.); lvbeibei2017@163.com (B.L.); wwenxuan_0522@163.com (W.W.); hehailin0306@163.com (H.H.); cwjian2001@163.com (C.J.); 13877173857@163.com (X.L.); 2 Key Laboratory of Deep Processing and Safety Control for Specialty Agricultural Products in Guangxi Universities, Education Department of Guangxi Zhuang Autonomous Region, Nanning 530004, China; 3 Institute of Biological Sciences and Technology, Guangxi Academy of Sciences, Nanning 530012, China

**Keywords:** bamboo shoot, health benefit, macro and micronutrients, quality and preservation, bamboo shoot processing residue

## Abstract

Bamboo shoots, as the young bamboo stems, are rich in protein, fiber, vitamins, and minerals, as well as many bioactive substances beneficial to health, and are gaining in importance worldwide as a healthy food and dietary supplement. However, fresh bamboo shoots lignify rapidly after harvesting and contain cyanogenic glycosides, limiting the safe and healthy consumption of bamboo shoots. To this end, based on the changes in nutritional composition and the physiological properties of fresh and post-harvest bamboo shoots, factors affecting the preservation of post-harvest bamboo shoots are emphasized, including a series of physical and chemical regimes and various processing methods for post-harvest preservation. Furthermore, a systematic biorefinery approach for using bamboo shoot processing residue to prepare value-added products is also discussed. Finally, the article also discusses issues related to sustainable development, safeguarding food security, and addressing potential health impacts in order to provide a scientific basis for researchers to further develop and increase the added value of bamboo shoots.

## 1. Introduction

Bamboo is a large herbaceous perennial and arborescent plant belonging to the bamboo subfamily [1]. Bamboo originated in China and is now widespread on every continent [2,3]. To date, over 130 genera and 1700 species of bamboo are widespread in tropical, subtropical, and temperate regions, especially during the rainy season [4]. Based on the Global Forest Resources Assessment 2010 Main Report, Bamboo forests cover 31.5 million hectares worldwide, around 1% of total forest area, with India and China having the largest bamboo forests, with 9.57 million hectares and 6.01 million hectares, respectively [5]. Due to its fast growth, rapid maturation, short overall production cycle, high biomass productivity, and adaptability, bamboo is regarded as one of the most versatile multi-utility forest tree grasses, providing humans with various living resources [6,7]. At present, bamboo has been widely employed in industrial applications such as papermaking, furniture, the building sector, fiber extraction, and biological raw material [8,9,10]. More importantly, owing to its rich variety of bioactive components, including flavonoids, polysaccharides, amino acids, phenolic acids, and volatile oils, the extracts of bamboo leaves exhibit multiple pharmacological activities [11] and are widely used as animal fodder, food, spices, and medicinal resources [11,12]. For example, bamboo leaves are rich in polyphenols, the extracts of which have been shown to have high antioxidant capacity against the 1,1-diphenyl-2-picrylhydrazyl radical, making it a potent natural phenolic oxidant that exerts anti-inflammatory, antimicrobial, antithrombotic, cardioprotective, and vasodilatory effects [13]. In addition, bamboo shoots are rich in phytosterols, which can be extracted for use as precursors for many pharmaceutical products, such as oral contraceptives, corticosteroids, anti-inflammatory drugs, metabolic steroids, and esterogenic hormones, among others [14].

Bamboo shoots are popular for their rich nutritional value and enjoyable flavor, with high demand every year. According to the National Bureau of Statistics of China, the total annual production of bamboo shoots in China reaches 1.03 million tons, with a total value of over USD 21.7 million [15]. The main export regions of canned bamboo shoots in China include Japan, the United States, Germany, the Netherlands, and South Korea. According to the General Administration of Customs of the People’s Republic of China, the largest number of canned bamboo shoots exported from China to Japan in 2021 was 64,415.75 tons, accounting for 48.7% of the total export volume. The quantity of canned bamboo shoots exported to the United States was 15,053.59 tons, accounting for 11.4% of the total export quantity. The quantity of canned bamboo shoots exported to Germany was 8601.47 tons, accounting for 6.5% of the total export quantity. The United States imports 30,000 tons of canned bamboo shoots annually from Taiwan, Thailand, India, and China for domestic consumption [16]. In Japan, the per capita consumption was only 1.2 kg in the 1950s and the current consumption of bamboo shoots is 3 kg per person per year [17]. At present, over two million tons of edible bamboo shoots are consumed in the world each year [17]. Statistic shows that some regions in northern India harvest more than 400 tons of bamboo shoots. By contrast, 26.68 million tons of bamboo shoots are harvested annually in Sikkim, Meghalaya, and Mizorah in northeastern India, of which 20 to 30 million tons are used for canned bamboo [18], creating great value for India [1].

Botanically, bamboo shoots are the aerial shoots or meristematic tissues of the bamboo, which are crisp and sweet and have an unparalleled flavor. Bamboo shoots have been consumed for more than 2500 years as part of local cuisine and traditional medicine [13,19,20,21]. According to the Compendium of Materia Medica, bamboo shoots have been used as traditional medicine for 2500 years [22]. Nowadays, due to the nutrients contained in bamboo shoots, including vitamins, minerals, phenols, phytosterols, and fiber, as well as their low fat and calorie content, they are also used as a health food, either as a fresh vegetable or processed into different forms [13,23]. More importantly, due to the high levels of bioactive substances, bamboo shoots have antioxidant, anti-diabetic, anti-obesity, anti-microbial, hypolipidemic, anti-inflammatory, and hypotensive activities, and they are used in a variety of medications in some countries to improve digestion, relieve hypertension, and prevent cancer and cardiovascular disease [1,24,25]. Therefore, bamboo shoots are fast becoming one of the world’s most popular foods, and global consumption of bamboo shoots exceeds 2 million tons per year [26]. China and Thailand are at the forefront of the international bamboo shoot sales market [19]. According to the General Administration of Customs of the People’s Republic of China in 2021, the number of canned bamboo shoots exported from China to Japan, the United States, and Germany was 64,415.75, 15,053.59 and 8601.47 tons, accounting for 48.7%, 11.4%, and 6.5% of the total export quantity, respectively [27].

In view of the increasing attention being paid to bamboo shoots, numerous studies have been conducted on the processing of the edible parts of bamboo shoots, their composition, and the health benefits of the extracted compounds [3,21,25,28,29]. Thus, this article aims to summarize the changes in nutritional composition and physiological properties of fresh and post-harvest bamboo shoots, factors affecting the preservation and processing strategies of post-harvest bamboo shoots, and the high value utilization of bamboo shoot processing residue, providing a systematic approach for researchers to further develop and increase the added value of bamboo shoots.

## 2. Nutrient Composition of Bamboo Shoots

**Figure 1:**
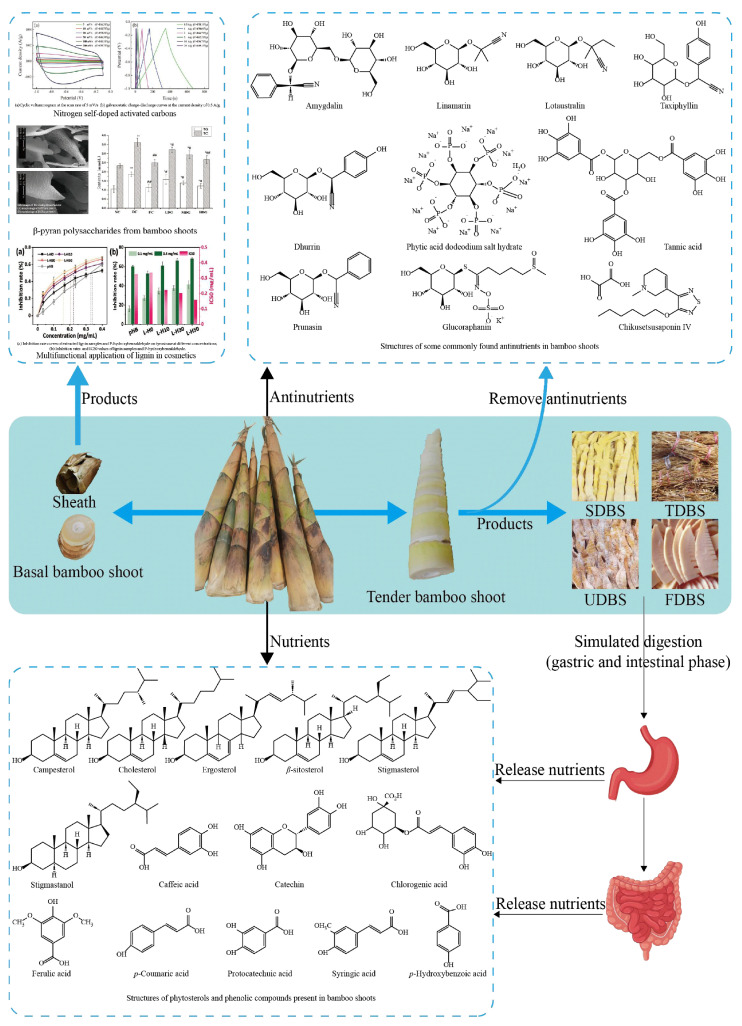
Structure, nutrients, and products of bamboo shoots [39,97]. * represents *p* < 0.05 compared with NC (normal group mice), which is significantly different; ** represents *p* < 0.01 compared with NC (normal group mice), which is significantly different (the degree of significance is greater than “*”); # represents *p* < 0.05 compared with DC group (mice in diabetic group) with significant difference; ## represents *p* < 0.01 compared with DC group (mice in diabetic group) with significant difference (the degree of significance is greater than “#”).

Generally, bamboo shoots are fresh plants (20–30 cm in length, narrow and pointed in shape, weighing more than 1 kg) with a soft, crisp, ivory-colored texture, which consist of a sheath, a stem (tip), and a basal shoot [19] (Figure 1). Normally, there are two types of bamboo shoots (winter shoots and spring shoots) available in a year, among which the bamboo shoots grown in spring are larger and tougher than those grown in winter [30], but the latter shoots have good taste and rich nutritional value [31]. However, the size and weight of bamboo shoots depend upon the location, depth and nutrition of the soil, irrigation and drainage conditions, climate, rainfall, temperature, and soil type and fertility [13,32,33].

More importantly, all species of fresh bamboo shoots contain a huge amount of moisture, a large number of volatile compounds (such as 2-pentylfuran, hexanal, benzaldehyde, 1-hexanol, and (E)-2-nonadienal [34]), are rich in protein, minerals, carbohydrates, and vitamins, but low in fat and cholesterol [35], and the nutrition and flavor vary depending on the species of bamboo [36]. Furthermore, bamboo shoots are also rich in bioactive substances such as phytosterols, polyphenols, polysaccharides, and fibers, which have antioxidant, lipid-lowering, prebiotic, anti-diabetic, and antimicrobial effects, and can aid in digestion, relieve high blood pressure, and prevent cancer and cardiovascular diseases [1,24]. Therefore, as they are low in calories (14–27 kcal 100 g^−1^) and rich in various nutrients, bamboo shoots are considered as a good source of food energy and an important health and functional food [37]. However, some bamboo shoots are not suitable for eating, such as *Bambusa balcooa*, *B.* (Wall. exMunro), *B. nutans* (Wall. exMunro), *B. tulda*, *Dendrocalamus brandisii* ([Munro] Kurz.), *D. hamiltonii*, *D. giganteus*, and *D. strictus* ([Roxb.] Nees.), and *Thyrsostachys siamensis*, which are a little bitter [3], and some species, such as *Phyllostachys edulis* (Carriere), *B. oldhamii* (Munro), *D. asper*, *D. latiflorus* (Munro), *P. makinoi* (Hayata), *P. pubescens*, and *T. siamensis* (Gamble), are the most commercially marketed shoots [38]. For example, China has 39 genera of bamboo plants and more than 500 types of bamboo industries according to the National Bureau of Statistics of China (NBS), in which more than 200 types of bamboo shoots are edible with good quality, such as *P. pubescens*, *D. latiflorus*, *P. praecox*, *P. pubescens*, *B. maydis*, *P. flavescens*, *D. squamosus*, and *P. grosvenoris*. The total annual output of bamboo shoots reaches 1.03 million tons, with a total value of more than USD 21.7 million [15].

### 2.1. Macronutrients

As shown in Table 1, bamboo shoots contain various macronutrients, like proteins, carbohydrates, amino acids, fiber, and fat, but their contents vary with the spices and different processing methods used [39].

#### 2.1.1. Proteins and Amino Acids

According to nutritional analysis, bamboo shoots are a potential source of high quality protein for humans [40]. However, there is no database for calculating the protein content of all bamboo shoot species [41], and the protein content is largely dependent on the type and maturity of the bamboo shoots, ranging from 1.49 to 4.04 g 100 g^−1^ wb and 21.1 to 33.4 g 100 g^−1^ db, respectively [5,13,42,43], which gradually decreases after heat treatment, canning, and fermentation. In addition, the protein content is not uniformly distributed in bamboo shoots, with a vertical distribution from tip to bottom [35]. For example, proteins from the bamboo shoot tip, base, and sheath were extracted using a deep eutectic solvent method, and the protein extraction rates were 3.92%, 1.55%, and 0.95%, respectively, with a molar ratio of choline chloride to acetylacetone of 1:6 [44]. More interestingly, the protein composition of bamboo shoots is mainly low-molecular-weight proteins (20.10–15.50 kDa) and histone-related proteins.

With regard to the composition of these proteins, bamboo shoots contain 17 amino acids, including threonine (Thr), serine (Ser), valine (Val), arginine (Arg), glycine (Gly), cysteine (Cys), alanine (Ala), methionine (Met), glutamic acid (Glu), leucine (Leu), histidine (His), aspartic acid (Asp), phenylalanine (Phe), isoleucine (Ile), lysine (Lys), proline (Pro), and tyrosine (Tyr), in which Tyr is the most abundant amino acid and Lys is the first limiting amino acid for most bamboo shoots [45]. In addition to their nutritional roles, free amino acids are also major components of bamboo shoot flavor and can be classified as sweet amino acids (Gly, Ala, Pro, Ser), bitter amino acids (Val, Ile, Leu, Tyr, Phe, Try), and fresh amino acids (Asp, Glu) based on their contribution to flavor. Among them, L-phenylalanine, L-ornithine, L-tryptophan, uridine, and adenine contribute the most to the flavor of bamboo shoots, while L-phenylalanine contributes the most to the bitter taste of bamboo shoots [46].

#### 2.1.2. Carbohydrates

With regard to total carbohydrates, polysaccharides (including starch, cellulose, and hemicellulose [47]), oligosaccharides (including sucrose, arabinoxylan trisaccharide, tetrasaccharide, and xyloglucan disaccharide [48]), and monosaccharides (such as fructose, glucose, and sucrose [49]) are abundant and available in multiple forms, with the content ranging from 2.0 to 9.94 g 100 g^−1^ in bamboo species [3]. More interestingly, an increase in the carbohydrate content is clearly observed during the storage of bamboo shoots, but the content decreases after fermenting and boiling in solutions with different salt concentrations. Recently, the carbohydrates in bamboo shoots have been extracted and extensively investigated as bioavailable macromolecules because of their antioxidant, immune-enhancing, anti-cancer, and many other activities [50].

Furthermore, as the “seventh nutrient”, most of the fibers in bamboo shoots are insoluble. Nutritional dietary fiber (NDF), acid detergent fiber (ADF), lignin, hemicellulose, and cellulose have been identified as the major fiber components, accounting for 60–90% of the total carbohydrates [5]. However, the fiber content and composition vary depending on the age and location of the bamboo shoots [49], critically determining the mouthfeel and taste of bamboo shoots [51]. Recently, chemical, enzymatic, and particle size distribution methods have been widely used to determine and improve the chemical and structural composition and physicochemical and functional properties of bamboo shoot fiber (BSF) [52,53,54]. For example, bamboo (*P. edulis*) shoot dietary fiber (BSDF-1) was extracted and its effect on the intestinal microbiota was explored with in vitro glycolysis. After a 48 h fermentation, the carbohydrate utilization rate and the total short-chain fatty acid content increased to 26.59% and 16.46 mmol/L, respectively, and correspondingly, the abundances of *Alistipes* and *Lactobacillus* were also increased, whereas those of *Escherichia-Shigella*, *Enterococcus*, and *Proteus* were significantly decreased, regulating the metabolism of the intestinal microbiota and the host [55].

### 2.2. Micronutrients

In addition to macronutrients, bamboo shoots are also rich in micronutrients, which are required in trace amounts by humans, and fresh bamboo shoots have good amounts of thiamine, niacin, vitamin A, vitamin B6, and vitamin E, but their levels depend on the species, age, different portions, and altitude of the bamboo shoots [42]. For example, rich levels of B vitamins are found in fresh bamboo shoots, which contain 0.09 mg 100 g^−1^ db of vitamin B1, 0.04 mg 100 g^−1^ db of vitamin B2, and 0.38 mg 100 g^−1^ db of vitamin B6 [24]. In addition, bamboo shoots are also rich in macronutrients [such as potassium (K), phosphorus (P), sodium (Na), calcium (Ca), and magnesium (Mg)] and microelements [including cobalt (Co), copper (Cu), nickel (Ni), manganese (Mn), selenium (Se), iron (Fe), and zinc (Zn)] [56], but the mineral content varies depending on the species, growth site, agroclimatic conditions, harvesting season, age, shoot part, sample preparation methods, and processing techniques [56,57]. For example, the contents of rubidium (Rb, 9%), sulfur (S, 11%), Mg (12%), Zn (46%), Ca (56%), and Fe (87%) in bamboo shoots were significantly increased after fermentation, and the contents of Na (18.79 g 100 g^−1^, dw), chlorine (Cl, 24.73 g 100 g^−1^, dw), and bromine (Br, 20.0 mg 100 g^−1^, dw) were also drastically increased after preservation in brine [56].

**Table 1:** Effects of different processing conditions on the nutrient composition of bamboo shoots.

Nutrient Composition	The Content in Bamboo Shoots	The Changes in Nutrient Composition	Refs.
**Macronutrients**			
**Protein**	Proteins in bamboo shoots consist of a diverse range of natural peptides, with the content ranging from 1.49 g to 4.04 g 100 g^−1^ (fresh bamboo shoots) and 1.8% to 25.8% (dry weight basis), respectively.	Protein content is attributed to species, growing site, climatic factors, age, cultivation, post-harvest processing conditions, and analysis method, but is decreased after boiling, cooking, storage, canning, and fermenting.	[13,58,59]
**Amino acid**	Ranging from 3.0 to 4.0% equivalent of leucine.	Bamboo shoots contain 17 different types of amino acids, including eight essential amino acids, but their levels are significantly decreased in pickled, old, fermented, canned, and boiled bamboo shoots.	[43,60]
**Carbohydrate**	Ranging from 4.32 to 6.92 g 100 g^−1^ fresh weight	Carbohydrate content is increased after the boiling process, but the soluble sugar content is decreased upon storage, extended fermentation, and boiling in salt solution.	[43,58]
**Organic acids**	Ranging from 3.3% to 5.2%	Oxalic, malic, and citric acids are the principal organic acid components, but their levels increase tremendously after fermentation.	[61]
**Fat**	Bamboo shoots are rich in non-polar lipids, glycolipids, and phospholipids, with a ratio of 17:27:56, and the main fatty acids are palmitic, linoleic, and linolenic acids.	Fat content (ranging from 0.3 g 100 g^−1^ to 3.97 g 100 g^−1^ wb) and composition are age-dependent and unevenly distributed in the tip, middle, and basal parts, but fat levels are reduced in boiled and steamed bamboo shoots, except for stir-fried bamboo shoots.	[3,49,60,62]
**Micronutrients**			
**Vitamin C**	Ranging from 3.0% to 12.9%, with the highest level in *D. hamiltonii* and lowest level in *D. sikkimensis* [61].	Vitamin C content is decreased after storage, fermentation, and canning.	[43]
**Vitamin E**	Ranges from 0.61% to 0.91%	Vitamin E content keeps decreasing in older and fermented shoots.	[43]
**Macroelements**			
**Potassium**	Potassium content ranges from 4190 to 6660 mg 100 g^−1^ of dry weight and is also affected by altitude, site, and different processing technologies.	Potassium content is significantly decreased after boiling, fermenting, brining, and different drying methods, among which freeze-drying is the most efficient method of retaining potassium content in shoots.	[56,57,63,64,65,66]
**Phosphorus**	Phosphorus content ranges from 460–930 mg 100 g^−1^ fresh bamboo shoots, with the highest content in *Phyllostachys manii*, but it os also affected by the site of the bamboo shoots.	Processing technique (boiled, brine-preserved, and fermented shoots) significantly impacts the concentration of phosphorus, in which the highest concentration is observed in sun-dried shoots.	[42,56,58]
**Magnesium**	Magnesium content ranges from 130 to 430 mg 100 g^−1^ fresh bamboo shoots, but is affected by bamboo species and site.	Magnesium content is slightly reduced after boiling and brine treatment, but slightly increases after fermentation and sun-drying.	[56,57,64,67]
**Calcium**	Calcium content ranges from 100 to 220 mg 100 g^−1^ fresh bamboo shoots but is affected by bamboo species and site.	Processing technique (soaked, brine-preserved, and boiled) significantly impacts the calcium concentration, which increases after processing in water-preserved shoots but is completely depleted after boiling for 25 min.	[56,57,58]
**Sulphur**	Sulphur is the third most abundant mineral in bamboo shoots, and its content ranges from 200 to 340 mg 100 g^−1^ in fresh bamboo shoots	Sulphur content is increased in shoots stored in water, sun-dried, and soaked but decreased in boiled, brine-preserved, and fermented shoots.	[56,57,58]
**Sodium**	Sodium content ranges from 10 to 90 mg 100 g^−1^, with the highest content recorded in D. membranaceous, and is affected by bamboo species, altitude, and site.	Sodium content is increased after processing in brine-preserved and fermented shoots.	[57,58]
**Chlorine**	Chlorine content does not vary significantly among species, ranging from 590 to 1680 mg 100 g^−1^.	Chlorine content is decreased after fermentation and boiling but increased drastically in brine-preserved shoots.	[57,67]
**Silicon**	Silicon content in fresh shoots ranges from 70–200 mg 100 g^−1^, containing over 70% organic silica.	Decline in silicon content after boiling and storage.	[68,69]
**Microelements**			
**Iron**	Iron content ranges from 4.7 to 25.8 mg 100 g^−1^ in fresh shoots but is affected by bamboo species, altitude, and site	Iron content is effectively retained in fermentation and sun-drying	[42,56,63,70]
**Zinc**	Zinc content is affected by bamboo species, altitude, and site, ranging from 6 to 21.07 mg 100 g^−1^.		[42,57,63]
**Copper**	Copper content is affected by bamboo species, age, and site, and the highest amount of copper is in *P. rubromarginata* (14 mg 100 g^−1^ in fresh shoots).	Copper content is remarkably decreased after fermentation and boiling but no difference in brine-preserved and older shoots.	[56,71]
**Manganese**	Ranging from 1.2 to 9.7 mg 100 g^−1^ fresh bamboo shoots, with the highest level recorded in *B. nutans*.	Manganese content is remarkably decreased after processing, in which fermentation, boiling, and brining can effectively retain the manganese content.	[56,64]
**Nickel**	Nickel content ranges from 0.7 to 1.2 mg 100 g^−1^, with the highest amounts in *C. capitatum* and *D. latiflorus*.	Nickel content is slightly decreased with the increase in bamboo shoot age but no difference after processing.	[43]
**Selenium**	In fresh bamboo shoots, the selenium content ranges from 0.0001 mg 100 g^−1^ (*B. nutans*) to 6.80 mg 100 g^−1^ (*D. hamiltonii*).		[72]

### 2.3. Bioactive Compounds

As shown in Figure 1, several phytochemicals, including carotenoids, phenolic compounds (flavonoids, phytoestrogens, phenolic acids), phytosterols and phytostanols, saponins, tocotrienols, organosulfur compounds (allium compounds and glucosinolates), and alkaloids, are present in bamboo shoots and have significant medical and nutraceutical applications [73]. For example, with the help of microwave-assisted extraction, polyphenols have been recovered from the shoots of *P. pubescens*, and its antioxidant properties greatly improve the elimination of DPPH, ABTS, and FRAP, paving the way for industrial applications in the production of functional foods [74].

#### 2.3.1. Phytosterols

Phytosterols are a class of secondary metabolites that belong to the family of lipophilic stanols and have a variety of health benefits, are capable of lowering serum cholesterol and preventing gastric ulcers, and also have anti-cancer, anti-inflammatory, and immunomodulatory effects [75]. Currently, several phytosterols have been found in bamboo shoots, among which β-sitosterol (24.6%), campesterol (2.2%), stigmasterol (1.2%), cholesterol (0.6%), ergosterol (0.2%), and stigmastanol (<0.1%) are the predominant phytosterols [13,76]. However, the content of phytosterols depends on the different processing methods, bamboo shoot type, part, and harvesting period. For example, spring shoots contain the highest level of β-sitosterol (83.33%), and winter shoots contain the highest level of cholesterol (3.4%), while the shoots harvested in summer contain the highest level of ergasterol (0.86%) [31]. More importantly, owing to the lipophilic nature of phytosterols, bamboo shoot oil extracted by supercritical carbon dioxide extraction was rich in phytosterols, with a total amount of 28.7 g/100 g^−1^, which was dominated by β-sitosterol (86%, *w*/*w*), providing an excellent candidate for the development of functional foods and nutraceuticals [77].

#### 2.3.2. Phenolic Compounds

In general, phenolics are mainly present as phenolic acids and flavonoids in bamboo shoots, among which eight phenolic compounds have been identified, namely, vanillic acid, protocatechuic acid, catechin, p-hydroxybenzoic acid, gallic acid, caffeic acid, butyric acid, ferulic acid, chlorogenic acid, and p-coumaric acid, among which protocatechuic acid, *p*-hydroxybenzoic acid, and butyric acid are the most common compounds [13]. As for flavonoids, the contents in bamboo shoots range from 279 to 1348 mg 100 g^−1^ DW (rutin equivalents, RE), among which orientin, isoorientin, isovitexin, vitexin, and tricin are mainly present in the insoluble form of free aglycone or flavonoid ligand [78]. However, the content of total phenols varies in the phenolic compounds present in bamboo shoots at different optimum harvesting times [79] and are increased during the storage process but effectively decreased by several treatments (such as soaking, boiling, and canning) except for fermentation [74,80], suggesting that consumption of fresh bamboo shoots could effectively retain their antioxidant properties and provide health benefits.

Currently, most studies have focused on total phenols and total flavonoids in raw bamboo shoot extracts [74,81], in which the major polyphenols are unstable and easily oxidized. In particular, some free phenolics are impaired by various factors during digestion [82]. Therefore, insoluble bound phenolics contribute more to antioxidant activity and bioavailability than free phenolics in vivo [83]. For example, the release pattern of bound phenolics in fumigated dried bamboo shoots (UDBS), steamed dried bamboo shoots (TDBS), salt-dried bamboo shoots (SDBS), and fermented dried bamboo shoots (FDBS) during simulated in vitro digestion was investigated. As a result, polyphenols in dried bamboo shoots (DBS) mainly existed in the form of bound phenolics, and the release of bound phenols from SDBS, TDBS, UDBS, and FDBS after gastrointestinal digestion amounted to 19.61%, 19.40%, 45.64%, and 19.66% of the total amount, respectively, which was significantly higher than the release after gastric digestion [84].

### 2.4. Anti-Nutrients

In addition to their nutritional value, fresh bamboo shoots contain a variety of anti-nutrients, including cyanogenic glycosides, glucosinolates, phytates, oxalates, saponins, and tannins, among which cyanogenic substances are the most predominant anti-nutrients, becoming a deterrent for consumption(Table 2) [85,86]. More interestingly, the content of cyanogenic substances varies with different stages of growth, different parts, bamboo species, geographical and climatic conditions, and soil conditions [87], ranging from 36.32 to 1717.85 mg kg^−1^ f.w [88]. However, cyanogenic substances (mainly cyanogenic glycosides such as axiphyllin) are highly unstable and responsible for an acrid taste and peculiar smell [89]. Therefore, various efficient processing and storage methods (fermentation, storage, drying, canning, steaming, roasting, blanching, boiling, and pickling) have been adopted to reduce cyanide levels while eliminating the bitter taste for safe and healthy consumption [5,90]. For example, more than 99% of cyanogenic glycosides are eliminated when bamboo shoots are boiled for 25 min, but this also significantly induces certain heat-labile reactions [91]. More importantly, low concentrations of anti-nutrients (such as phytate, lectins, tannins, amylase inhibitors, and saponins) might also have beneficial health effects by reducing blood glucose, insulin responses, and cancer risks [92]. For example, saponins have a variety of health benefits, including immunostimulatory and cholesterol lowering effects, and prominent resistance properties, including anti-tumor, anti-inflammatory, and anti-viral activities, as well as anti-fungal and anti-parasitic activities [93].

In summary, owing to their high nutrient and medicinal value, bamboo shoots are not only a traditional food ingredient but are also used in folk medicine for the treatment of dysentery, diarrhea, diabetes, influenza, and infection [94], and they have attracted considerable attention [95,96]. For example, the protective effect of bamboo shoots against high-fat diet-induced gut dysbiosis in mice was investigated following 12 weeks of feeding in C57BL/6J mice. As a result, bamboo shoot supplementation was effective in reducing body weight by 30.56% in obese mice while liver damage, insulin resistance, and inflammation were reduced, suggesting that bamboo shoots were effective in reducing obesity in mice. In addition, bamboo shoots increased the levels of short-chain fatty acids (SCFAs) and SCFA-producing bacteria (e.g., Lachnospiraceae_NK4A136_group, and Norank_f_Muribaculaceae) while decreasing the levels of harmful bacteria (e.g., Blautia and Burkholderia-Paraburkholderia), suggesting that bamboo shoots were effective in increasing a wide range of beneficial fecal metabolites, thereby altering the gut microbiota [96].

**Table 2:** Anti-nutrients in bamboo shoots.

Anti-Nutrients Composition	The Content in Bamboo Shoots	The Changes in Nutrient Composition	Refs.
**Taxiphyllin**	Bamboo shoots contain high amounts of cyanogen glycoside, and the maximum concentration is at the shoot tip.	Taxiphyllin is highly unstable and easily decomposed in boiling water.	[89,98]
**Glucosinolates**	Levels of glucosinolates of all species vary slightly and range from 26.45 to 29.99 mg 100 g^−1^ f.w.	Drying, fermentation, boiling, and soaking can significantly reduce the total glucosinolate content.	[85,99]
**Oxalates**	Oxalate content depends on different species and ranges from 112.2 to 462.0 mg 100 g^−1^ f.w.	All processing treatments cause a decrease in the oxalate content of fresh shoots, among which fermentation and freeze-drying are the best techniques for removing oxalates from bamboo shoots.	[5,85,100,101]
**Saponins**	Ranging from 229.58 to 246.22 mg 100 g^−1^ f.w.	Drying, fermentation, boiling, and soaking can significantly remove saponin.	[102]
**Phytate**	Phytate content in the fresh shoots ranges from 86 (*B. tulda*) to 97 mg 100 g^−1^ f.w. (*D. membranaceous*).	Fermentation, boiling, storage, and soaking can significantly remove phytate, but drying has no effect on phytate, and 100% reduction in phytate was obtained after six-month storage.	[85,101,102]
**Tannins**	Tannins, being polyphenolic compounds, are heat labile and water soluble, and their content ranges from 31 to 51 mg 100 g^−1^ f.w.	Boiling, fermentation, and soaking can effectively remove tannins but drying has no significant effect on tannins.	[85,100]

## 3. Preserved/Stored Bamboo Shoots: Quality and Safety Aspect

**Figure 2:**
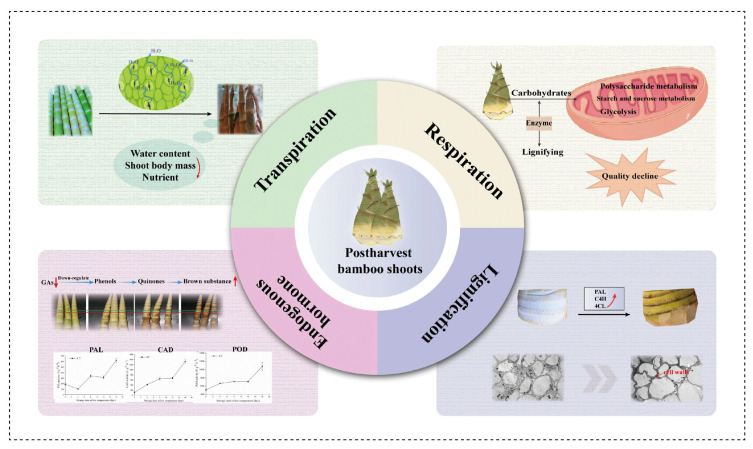
Physiological changes of post-harvest bamboo shoots [97,103,110].

Generally, the roots of bamboo shoots are severely wounded after harvesting them with shovels or hoes, making bamboo shoots prone to disease, including physiological diseases (such as cold damage, browning, and senescence) and pathological diseases, due to mechanical injury [103]. Furthermore, as the most metabolically active part of bamboo, post-harvest bamboo shoots are still an active organic mass performing various physiological activities, including respiration, transpiration, and aging, owing to the high moisture content, making bamboo shoots susceptible to water loss, lignification, and microbial infection during picking, transportation, and storage (Figure 2) [104]. Therefore, high respiration and microbial spoilage are the main factors affecting shelf-life, quality, and nutrient changes in post-harvest bamboo shoots [105,106], making their shelf-life generally less than 3 days at room temperature and up to 7 days above 8 °C, thus affecting consumer preferences and limiting long-distance transport [107,108]. As the market demand for processed bamboo shoots continues to increase, it is necessary to have a comprehensive understanding of the basic physiological characteristics of bamboo shoots after harvesting and then develop processing techniques to preserve bamboo shoots in a form that maintains stable quality and ensures demand for bamboo shoots during the off-season [19].

### 3.1. Quality Changes of Post-Harvest Bamboo Shoots

Owing to the combined actions of metabolism and microorganisms, the appearance of post-harvest bamboo shoots will change in relation to the following aspects: (1) Color and form: after harvesting, the water content in bamboo shoots will continuously decrease, causing tissue and organ atrophy and a dry, brown, and moldy state [104]. The moisture content of fresh shoots is extremely high, reaching 90% by weight, while on the fourth day, the moisture was reduced to 86.28% when stored at 18 °C [66]. In addition, after exposure to the air, phenolic substances are rapidly oxidized, and a large amount of quinone accumulates, which is further oxidized and polymerized to form black substances [109], changing the color of bamboo shoots from white to yellowish, dark yellow, and finally dark brown with extended storage time. (2) Texture: due to microbial infection of the damaged surface, the basal part of bamboo shoots becomes soft and sticky [110]. In addition, the actions of strong respiration and transpiration can also effectively increase the degree of fibrosis, decreasing the commercial value of bamboo shoots. (3) Nutritional quality: owing to the absence of light and nutrient supply, the contents of protein, soluble sugars, VC, soluble solids, and other nutrients in bamboo shoots are changed. In bamboo shoots, the protein content decreased from about 5 g kg^−1^ to about 1 g kg^−1^ and the soluble polysaccharide content decreased from about 140 g kg^−1^ to about 30 g kg^−1^ after post-harvest storage at room temperature for 12 d [110]. The content of VC of bamboo shoots was changed from 3 g kg^−1^ to 4.5 g kg^−1^ after storage at room temperature for 60 d [111], which may be related to the maturation process of bamboo shoots after harvesting, but the content of VC decreased in light-avoidance conditions [112]. Additionally, the contents of cellulose and lignin increased rapidly from the cut to the top, the lignin content of the top increased from 7.92% to 19.05% [110].

### 3.2. Physiological Changes of Post-Harvest Bamboo Shoots

#### 3.2.1. Endogenous Hormone Changes

During the storage of post-harvest bamboo shoots, the endogenous hormones change, and the content of gibberellins decreases rapidly in the early stage of storage and remains stable after 15 days. Furthermore, the indoleacetic acid content is stable in the early stage of storage but decreases rapidly after 10 days, while the content of abscisic acid decreases rapidly after 15 days and then increases slightly at the end of storage [104]. More importantly, the changes in endogenous hormones are closely related to the activities of phenylalanine ammonia lyase (PAL), peroxidase (POD), and PPO, and the change in hormone ratio affects the lignification of bamboo shoots. For example, as a natural endogenous plant hormone, ethylene is produced under various environmental stresses (such as injury stress, low temperature, pathogen attack, chemical treatment, etc.), and is involved in chilling damage and the lignification process of bamboo shoots [104]. Therefore, inhibition of ethylene biosynthesis or its effect plays an important role in alleviating cold damage and prolonging the storage life of bamboo shoots [113].

#### 3.2.2. Lignification

Bamboo shoots undergo a rapid lignification process after harvesting, even during storage in a cold chamber, which causes rapid hardening of bamboo shoots and severely reduces the palatability of fresh bamboo shoots [114,115], making lignification the largest problem in the bamboo shoot industry. Previous studies have published the genome of bamboo shoots, providing information on potential candidate genes to speculate the molecular basis of the physiological process and the coordinated molecular mechanism of secondary wall thickening and lignification of bamboo shoots after harvest [103,116]. Generally, the contents of lignin and cellulose determine the degree of lignification of bamboo shoots. Lignin is a complex phenylpropanoid polymer synthesized by the polymerization of three monolignin monomers (guaiacyl, butyryl, and hydroxyphenyl) with peroxidase enzyme and, during cold storage, the levels of lignin and cellulose increase, accompanied by increases in PAL, cinnamyl alcohol dehydrogenase (CAD), and POD activities [108]. Furthermore, the mechanism of lignification in post-harvest bamboo shoots during room temperature storage has also been elucidated. It was found that the lignification of bamboo shoots is closely related to the increase in POD activity, the increase in the expression levels of the *MYB20*, *MYB43*, *MYB63*, and *MYB85* genes, and the decrease in the expression levels of the *KNAT7* and *NST1* genes, and the lignification process of bamboo shoots of different varieties differs significantly [117].

### 3.3. Factors Affecting the Preservation of Post-Harvest Bamboo Shoots

Generally, temperature is the most crucial factor affecting the respiration rate of post-harvest bamboo shoots, and low temperature can effectively inhibit respiration, reduce the loss of total sugars and acid, the contents of lignin and cellulose, as well as the activities of PAL, CAD, and POD, and is conducive to bamboo shoot storage [108]. However, bamboo shoots are sensitive to low temperatures and are susceptible to freeze injury (CI) symptoms, such as flesh browning and the appearance of mold. Therefore, the incidence of CI was used as a factor for evaluating post-harvest storage potential in long-term cold storage [118]. For example, dipping in 0.07 mM sodium nitroprusside (SNP) prior to storage at 1 °C can effectively reduce the incidence of CI in cold-stored bamboo shoots by activating the metabolism of polyamines, γ-aminobutyric acid, and proline, enhancing the chilling tolerance of bamboo shoots, but, being a chemical method, it can make them commercially difficult [106].

Furthermore, moisture is an important factor in maintaining the physiological activity of bamboo shoots and also an indicator of their freshness. Loss of water can effectively increase the saccharin content of bamboo shoots, and enzyme activity will also be improved, which makes bamboo shoots easily hydrolyzed, infected, and decayed. In addition, bamboo shoots under low-light conditions have a mild bitter flavor, while bamboo shoots under shaded conditions have a heavier bitter flavor. The phenolic acid content related to the tyrosine metabolism pathway was downregulated under the shaded treatment, indicating that shading could reduce the bitter flavor of bamboo shoots. The phenolic acid content associated with the tyrosine metabolism pathway was downregulated under the shaded treatment, which indicated that shading reduced the accumulation of phenolic acid, which in turn conveys the bitter flavor of bamboo shoots. The accumulation of phenolic acids also contributes to the bitter flavor of bamboo shoots [119]. Therefore, temperature, humidity, enzyme activity, and water content are factors that affect shoot respiration and need to be considered as factors in the development of post-harvest storage systems with the aim of extending the shelf life of post-harvest bamboo shoots [114,117]. For example, low temperature (up to −50 °C) can effectively limit the enzymatic, microbial, and chemical reactions in bamboo shoots, thereby preventing excessive moisture loss and maintaining a decreased respiration rate (<4.08 mmol CO_2_ kg^−1^ h^−1^ at 20 °C) [110]. In summary, owing to the complexity of bamboo shoots, there is still a gap in creating methods that inhibit the rapid post-harvest senescence of bamboo shoots and stabilize the quality of the product. At present, a suitable preservation system for fruits and vegetables is essential in order to: (a) prevent or delay microbial decomposition through sterile handling, filtration, drying, freezing, or heating; (b) prevent or delay self-decomposition of the product; and (c) prevent damage caused by poor handling, insects, etc. For bamboo shoots, an effective storage system can be developed with low temperature and high humidity, or low oxygen and high carbon dioxide, or low ethylene and asepsis, which are beneficial for bamboo shoot preservation [120].

### 3.4. Advances in Bamboo Shoot Preservation and Storage

In addition to the complexity of bamboo shoots, it is important to consider that most bamboo shoots are sourced from mountainous areas over long distances and transportation may take several hours. To this end, various forms of processing and pretreatment have been adopted to prolong the preservation of bamboo shoots for an annual supply (Table 3). Currently, chemical preservation is the most widely used method to inhibit rapid post-harvest aging, ripening, hardening and lignification of fresh shoots [114]. Several chemicals (such as oxalic acid, salicylic acid, nitric oxide [121], 1-methylcyclopropene (1- MCP) [122], chitosan [123], and melatonin [115]) have been used to impregnate or saturate coated or uncoated bamboo shoots to maintain post-harvest quality by balancing the formation and elimination of reactive oxygen species, inhibiting the activity of enzymes involved in lignin and cellulose biosynthesis and altering metabolism.

However, owing to food safety considerations, consumers are more receptive to physical preservation methods to improve the shelf-life of post-harvest bamboo shoots [124]. Thus, natural preservatives or physical and non-contact preservation methods have attracted considerable attention [125], and several chemical treatments (such as antioxidants, chelating agents, antimicrobial compounds, enzyme inhibitors [123]) and physical treatments [such as high hydrostatic pressure [126], hot air [127], ultraviolet-C exposure (254 nm), ozone treatment [128,129], hypobaric atmosphere, irradiation [124], gamma radiation [130], and modified atmosphere packaging (MAP) [131] have been developed to maintain the quality of freshly-cut bamboo shoots [124].

Recently, to obtain better preservation, the combination of chemical and physical treatments was also adopted to maintain the post-harvest quality of bamboo shoots [123]. For example, *Lactobacilli* and *Cyanobacteria* were significantly inhibited during combined melatonin and UV-C treatments, and important metabolites corresponding to lignin content and resistance, including precursors of secondary metabolites associated with phenylpropanoid biosynthesis (Tyr, Leu, and Phe), were accumulated. More importantly, UV-C exposure upregulated 19 of 22 fatty acyl groups, whereas melatonin treatment increased the levels of 12 of 16 flavonoids, including lignin and butane 4′-arabinosyl-(1->4)-galactoside [125]. However, packaging and cooling operations can control these parameters, wherein the optimal storage temperature and the rate at which the product is brought down to the storage temperature have strong influences on the shelf-life of bamboo shoots. Therefore, the selection of the right preservation technology is very important for the quality and shelf-life of the product, and the development of a suitable system requires the integration of the chosen packaging material (bag or microporous low-density polyethylene film), the MAP (2% O_2_, 5% CO_2_, 93% N_2_), and the optimal temperature for preserving bamboo shoots [132].

**Table 3:** Effects of different processing methods on the post-harvest storage of bamboo shoots.

Handling Method	Operating Conditions	Effect Analysis	Refs.
**Physical regimes**			
**Heat treatment**	At 45 °C for 5 h was beneficial for fresh shoot storage	Effectively inhibited disease development and respiration, delayed ethylene production, and delayed tissue lignification	[127]
**Ultraviolet-C exposure (254 nm)**	At 4.24 kJ m^−2^ and 20 °C for 2 d At 4.0 kJ m^−2^ at 6 °C	Significantly inhibited pulp strength, respiration rate, weight loss, wound browning, disease development, and cellulose and lignin synthesis, which in turn greatly delayed the development of greenness and toughness and increased total phenol concentrations.	[133]
**gamma radiation**	A pretreatment with a dose of 3 kGy at 4 °C	Inhibited the activities of PAL, POD, and PPO, and then retarded the increase in the levels of lignin and cellulose, preventing the lignification and browning processes and slowing the degradation of soluble proteins and sugars.	[124]
	A pretreatment with a dose of 0.5 kGy at 2 °C	Effective in reducing cold damage, ethylene production, lignin buildup, and rot.	[130]
**Hypobaric storage**	At 50 kPa at 2 °C At 600 MPa at 25 °C	Effective inhibition of resistance development and lignin and cellulose accumulation in new shoots.	[126,134]
**High hydrostatic pressure (HHP) processing**	300 MPa for 10 min	At the end of storage, weight loss and increase in titratable acidity (TA) were minimal. Respiratory strength, appearance, and color of bamboo shoots were stabilized, providing an effective method to maintain the quality of bamboo shoots stored at room temperature.	[135,136]
**Chemical regimes**			
**Melatonin**	With a pretreatment of dipping in 1.0 mM melatonin and storage at 4 °C	Effectively retarded lignification and significantly reduced hardening, yellowing, and biosynthesis of lignin and cellulose.	[115]
**Diphenyliodonium iodide**	Pretreatment of 5 mmol diphenylammonium iodide and storage at 20 °C	Displayed lower values of firmness and lignin content.	[114]
**Brassinolide**		Reduced frost damage to bamboo shoots by increasing the activity of enzymes related to energy and proline metabolism.	[137].
**Oxalic acid**	With a pretreatment of 10 mM oxalic acid for 10 min and storage at 6 °C	Oxalic acid treatment improved the integrity of bamboo shoot membranes and reduced respiration while decreasing total sugar content and weight loss, reducing disease incidence, inhibiting enzymatic browning, and slowing down lignification during cold storage.	[114]
**Sodium nitroprusside**	Dipping in 0.5 mM sodium nitroprusside (a nitric oxide donor) and storage at 20 °C or 10 °C	Successfully prevented increases in firmness, lignin and cellulose accumulation, and external browning.	[138]
**Salicylic acid**	With a pretreatment of 1.0 mM salicylic acid and storage at 1 °C	Suppressed chilling injury and flesh browning.	[127]
**1-MCP and SO_2_ treatment**		It effectively inhibited the physiological metabolism of bamboo shoots, reduced browning and fiber lignification, maintained good quality during storage, and slowed down the aging of bamboo shoots.	[111]
**UV-B treatment**	At a dose of 8.0 kJ m^−2^ and then stored at 6 °C along with 85–90% relative humidity (RH) for 15 d	Apparently slowed down increased rates of flesh firmness, weight loss, and contents of cellulose and lignin. It also decreased the activities of 4-coumarate CoA ligase, peroxidase, cinnamyl alcohol dehydrogenase, and phenylalanine ammonialyase during cold storage.	[139]
**Exogenous hydrogen peroxide (H_2_O_2_) and diphenyliodonium iodide (DPI)**	Soaking in 10 mM H_2_O_2_ or 5 mM DPI for 10 min, storage at 20 ± 1 °C for 12 d.	H_2_O_2_ treatment accelerated the accumulation of endogenous H_2_O_2_ by activating NADPH oxidase, while DPI treatment inhibited NADPH oxidase activity, leading to decrease in endogenous H_2_O_2_ levels.	[114]
**Melatonin and UV-C treatments**	Freshly cut bamboo slices were immersed in 1.0 mM melatonin solution for 5 min and exposed to a UV-C lamp (75 W) at 30 cm above the sample tray for 5 min, and then stored at 25 °C, 90% RH for 7 d.	Effectively maintained the quality of fresh-cut bamboo shoots during storage by altering microbial diversity and metabolites.	[125]
**Preservation system**			
**Hydro-cooling**	Vacuum-cooling combined with hydrocooling and vacuum-drying processes	Advantages of a lower number of bacteria, higher stability, longer preservation period, and better appearance in low-temperature storage but required higher equipment costs and higher losses of soluble solids and ascorbic acid of bamboo shoots.	[132]
**Modified atmosphere packaging (MAP) System**	Storing bamboo shoots with 0.04 mm thick LDPE bag, 2% O_2_, 5% CO_2_, and 93% N_2_.	Significantly inhibited the lignification and browning of bamboo shoots.	[140]

### 3.5. Processing Strategies of Bamboo Shoots

**Figure 3:**
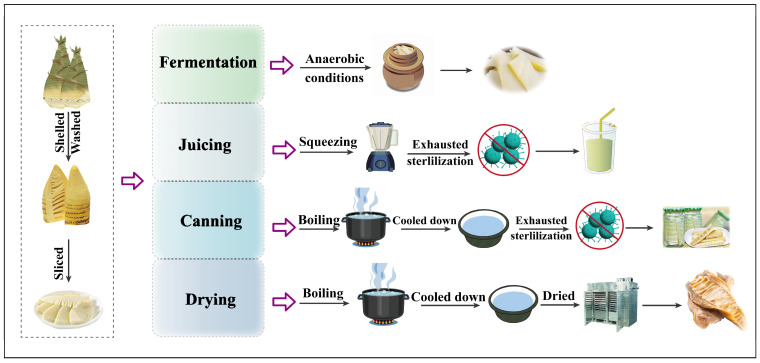
Processing workflows of bamboo shoot products [5,153].

The above storage methods can maintain the quality of fresh bamboo shoots after harvest for a period of time and extend the shelf-life of the product, but ensuring the supply of bamboo shoots throughout the year is still a problem. In addition, another factor that discourages people from consuming bamboo shoots is the anti-nutrients contained in bamboo shoots [22]. Thus, eating fresh raw bamboo shoots is not recommended, and several processing methods have been adopted to effectively reduce anti-nutrient levels and improve the nutritional value, sensory properties, and functional qualities of bamboo shoots, satisfying global market transactions (Figure 3) (Table 4) [28,141,142,143]. Correspondingly, a series of bamboo shoot products, including canned bamboo shoots, boiled bamboo shoots [144], fermented bamboo shoots, dried bamboo shoots [3,137], pickled bamboo shoots, bamboo shoot powder, bamboo shoot juice, bamboo desserts, and bamboo beer (prepared from bamboo culms) can be found in China, Japan, Thailand, and Malaysia [145,146,147]. For example, *Lactobacillus plantarum* was utilized to ferment red dates and bamboo shoots to produce a beverage. The nutrient content of the fermented beverage was increased, and more importantly, the antioxidant index was also improved, with the total phenolic content, total antioxidant capacity, and superoxide anion scavenging capacity increased by 11.09%, 12.30%, and 59.80%, respectively [147].

However, although the above treatments were effective in preserving bamboo shoots and prolonging their availability throughout the year, the physical and chemical changes associated with the treatments also altered the nutritional integrity of bamboo shoots (Table 3) [45,148]. For example, canned bamboo shoots can be used directly as a raw material for preparing dishes and processing other downstream products, and they can be stored at room temperature for 2 years and further processed into various products according to market demand [27], but there is an overall decrease in the nutrient components, except dietary fiber, especially vitamins and minerals, during canning [149]. Similarly, the contents of fiber, minerals, flavor substances, and cellulose and antimicrobial activity were effectively increased due to the presence of lactic acid bacteria and yeast, resulting in fermented bamboo shoots having a variety of probiotic effects, such as lowering cholesterol levels, antioxidant, anti-free radicals, anti-aging, pre-immunity, anti-cardiovascular disease, anti-cancer, and so on [150]. More importantly, fermentation can also effectively reduce bamboo shoot toxicity, in which the levels of taxiphyllin, p-hydroxybenzaldehyde and methyl-p-hydroxybenzoate are decreased, they are transformed into intermediate compounds (p-hydroxybenzoic acid and p-hydroxybenzyl alcohol) and then finally converted into p-hydroxytoluene during fermentation [86]. However, fermentation can also significantly decrease the hardness, chewiness, elasticity, and cohesiveness of bamboo shoots [151,152].

**Table 4:** Effects of different processing methods on the nutrient composition of bamboo shoots.

Processing Methods	The Changes in Nutritional Integrity of Bamboo Shoots	Product	Refs.
**Soaking**	Overnight soaking is an effective precooking method for removing the acrid taste and smell from shoots, but its effects depend on the temperature, time, and soaking medium.	Clear water bamboo shoots	[88]
**Boiling**	Boiling with different concentrations of salt can effectively reduce cyanogen (a great reduction of 92.53–96.62% cyanogens), protein, and sugar contents.	Boiled bamboo shoots	[154]
**Canning**	Canning is a commonly used method of heat inactivation of microorganisms in hermetically sealed packaged products, but it reduces nutrients other than fiber. In order to reduce the sterilization temperature to protect the color and keep food safe during its shelf-life, dilute citric acid solution (about 0.15% (m/m) and preservatives (potassium sorbate and sodium benzoate) can be added to packages to ensure that the shelf-life of the food exceeds 12 months at room temperature [151].	Canned bamboo shoots	[155]
**Fermentation**	Fermentation can effectively decrease levels of nutrient components, except phenols, flavonoids, and dietary fiber, but provides bamboo shoots with tremendous health benefits, like anticancer, antioxidant, anti-aging, cardioprotective, weight loss, and probiotics.	Ulanzi, Naw-mai-dong	[151]
**Pickling**	Salt content, pickling temperature, bleaching time, and CaCl_2_ content have significant effects on the hardness of pickled bamboo shoots.	Salt-dried bamboo shoots	[156]
**Osmotic dehydration**	Before drying, bamboo shoots were soaked in 50 °B sucrose syrup and 10% salt using temperature and time of 40 °C and 90 min, respectively, achieving better product quality in terms of rehydration ratio, color, texture, etc.	Seasoned bamboo shoots	[157]
**Drying**	Drying (oven-drying, sunlight-drying, freeze-drying, superheated steam-drying, solar-drying, hot air-drying, microwave-drying, and combined drying) effectively reduces water activity, inactivates enzymes, and inhibits the growth of microorganisms. Freeze-drying treatment seriously destroys the texture of fresh bamboo shoots, giving them a reticulated sponge structure with poor water retention and palatability, while freeze-drying has a better effect on the retention of nutrients and the rehydration rate.	Dried bamboo shoots	[91,158,159]

## 4. Current Management of BSPR

**Figure 4:**
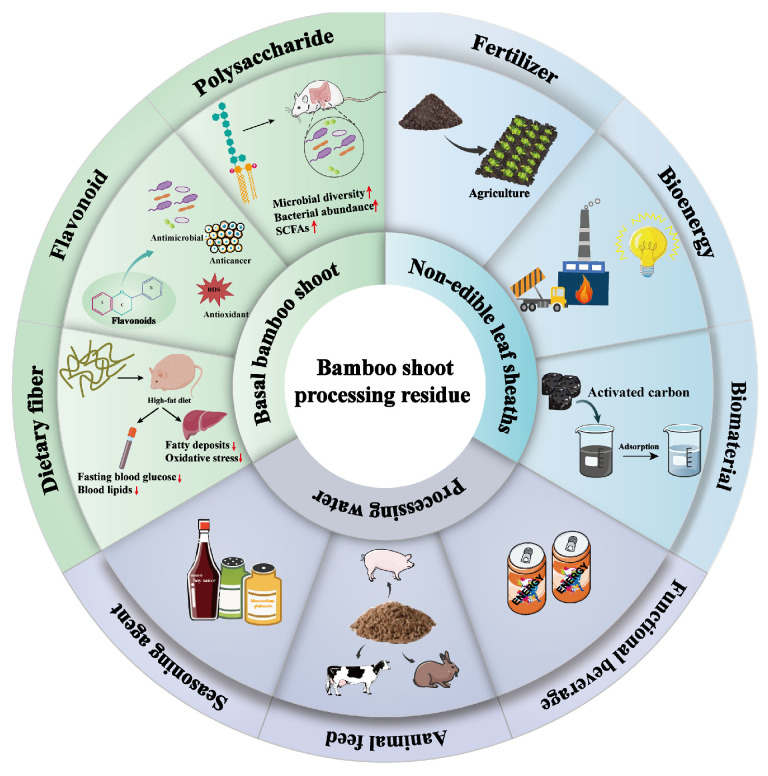
Biorefinery of bamboo shoot processing waste residue.

The traditional processing of bamboo shoots generates up to 70% of byproduct waste, which is partly thrown away as waste and only 30% is consumed [160]. As shown in Figure 4, bamboo shoot processing residue (BSPR) can be broadly categorized as consisting of inedible leaf sheaths, bamboo shoot bases, and processing water [161]. However, most BSPR is eliminated from the environment through composting, diameter burning, or landfilling [162], or converted to traditional products of low-added value, such as handicrafts and toys, door curtains, footwear, flooring materials and soles, hats, furniture, fertilizer and animal feed, and disposable tableware [163,164,165]. In fact, BSPR contains various types of bioactive components, exhibiting potential nutraceutical effects for functional food products, nutraceuticals, and cosmetics [39,166,167]. Thus, a systematic approach to biorefining is necessary to maximize the value of the waste.

With regard to the basal part of bamboo shoots, it is a new resource that can be used to produce flavonoids, carbohydrates, and fiber [52,167,168]. For example, the levels of flavonoids in bamboo shoots increased from 1.39% to 37.34% during optimal adsorption/desorption and PI3K, AKT, and IRS-1 protein expression was regulated via the PI3K/AKT pathway, resulting in potent hypoglycemic activity [169]. Similarly, feeding mice with BSPR, a polysaccharide extracted from the roots of bamboo shoots, was found to significantly increase their body weight, along with a decrease in pH and an increase in the concentration of SCFAs in the feces, compared to the control group. In addition, BSPR affected the composition and diversity of the intestinal microbiota, especially increasing the number of beneficial bacteria such as *Bacteroides*, *Lactobacillus*, and *NK4A136_group Lactobacillus* [54].

Furthermore, as the major byproduct of bamboo shoots, bamboo shoot shell (BSS), which covers the shoots, is black, brown, yellow, or purple, and its dry weight consists of approximately 20–48% cellulose, 20–36% hemicellulose, 2–18% lignin, 7% pectin, 13% fat, and other substances [170,171]. Therefore, BSS is also a potential biotechnological source and is sustainably used as a cheap dietary ingredient [171]; feedstock for bioenergy and biomaterials [172]; for the extraction of nanocellulose [173], carbon-based carriers [174], polysaccharides [175], and bioactive compounds [176]; and in the cultivation of edible mushrooms [177]. For example, ultrasound combined with phosphotungstic acid hydrolysis yielded two fractions, BSSP-A and BSSP-B, which were isolated after elution on a DEAE Sepharose fast-flow column and continuous NaCl solution. It was found that among these fractions, BSSP-B exhibited better protection against H_2_O_2_-induced oxidative damage in WRL-68 cells, which was achieved through protection of the cell membrane structure, maintenance of mitochondrial membrane potential, and increasing intracellular antioxidant enzyme activities [53].

The processing water from commercial processing of bamboo shoots is a major waste stream: about 2 t of wastewater is generated per ton of bamboo shoots during cooking, soaking, and cooling [178]. However, owing to the large amounts of peptides and amino acids in the wastewater, the processed wastewater has the potential to be used to produce protein functional beverages, supplements, and flavors. For example, fermented bamboo shoot processing waste (FBSPW) can be used in feed processing, and 12% of FBSPW added to weaned piglet feed was found to improve nitrogen and lipid metabolism as well as the intestinal microflora in weaned piglets, resulting in a significant reduction in the daily feed intake of weaned piglets as well as reductions in the serum triglyceride level and urea nitrogen level [179].

## 5. Conclusions and Future Perspective

As a healthy and nutritious food, bamboo shoots are gaining popularity all over the world for their high nutritional value, health benefits, and low environmental impact. Bamboo shoots are widely used in the production of a variety of high-quality products, such as pickles, sweets, nuggets, crackers, chutneys, noodles, and namkeen [153]. Therefore, bamboo shoots have the potential to add economic activities at industrial and society levels through cultivation, processing, packaging, and commercialization. However, their short shelf-life and several anti-nutrients (such as cyanogenic glycosides, glucosinolates, phytates, oxalates, saponins, and tannins) affect the bioavailability of nutrients and micronutrients, which can lead to nutrient deficiency and malnutrition [180]. Therefore, there is a need to combine indigenous knowledge with modern scientific knowledge to develop appropriate processing and preservation methods to reduce the levels of anti-nutrients in bamboo shoots, in order to provide safer food and to increase consumers’ enjoyment of bamboo shoots [5,181]. In the future, the focus should be directed to the following aspects for sustainable development of a bamboo shoot-based food industry.

- With regard to anti-nutrients, further research should be conducted on innovative methods to effectively identify and select the cultivation of suitable edible bamboo shoot varieties with low cyanogenic glycoside content. Elimination of toxic cyanogenic glycosides ensures food safety while maintaining the nutrients and bioactive substrates in bamboo shoots.
- In view of the beneficial health effects of anti-nutrients at low concentrations, the mechanisms underlying the nutritional composition and therapeutic effects of bamboo shoots in regional medications should be completely understood and then explored as an advanced medicinal product or healthy diet to solve a series of diseases.
- A more systematic approach to biorefining is needed, with the goal of increasing the value of BSPR as a value-added product.

## Data Availability

No new data were created or analyzed in this study. Data sharing is not applicable to this article.

## References

[1] Basumatary A., Middha S.K., Usha T., Basumatary A.K., Brahma B.K., Goyal A.K. (2017). Bamboo shoots as a nutritive boon for Northeast India: An overview. 3 Biotech.

[2] Chauhan O.P., Unni L.E., Kallepalli C., Pakalapati S.R., Batra H.V. (2016). Bamboo Shoots: Composition, Nutritional Value, Therapeutic Role and Product Development for Value Addition. Int. J. Food Ferment. Technol..

[3] Singhal P., Bal L.M., Satya S., Sudhakar P., Naik S.N. (2013). Bamboo Shoots: A Novel Source of Nutrition and Medicine. Crit. Rev. Food Sci..

[4] Yeasmin L., Ali M.N., Gantait S., Chakraborty S. (2015). Bamboo: An overview on its genetic diversity and characterization. 3 Biotech.

[5] Wang Y., Chen J., Wang D., Ye F., He Y., Hu Z., Zhao G. (2020). A systematic review on the composition, storage, processing of bamboo shoots: Focusing the nutritional and functional benefits. J. Funct. Foods.

[6] Goyal A., Sen A. (2016). In vitro regeneration of bamboos, the “Green Gold”: An overview. Agric. Food Sci. Environ. Sci..

[7] He M.X., Wang J.L., Qin H., Shui Z.X., Zhu Q.L., Wu B., Tan F.R., Pan K., Hu Q.C., Dai L.C.J.C.P. (2014). Bamboo: A new source of carbohydrate for biorefinery. Carbohydr. Polym..

[8] Paul D., Gaff M., Tesarova D., Hui D., Li H.T. (2023). Recent advancements in nanotechnology application on wood and bamboo materials: A review. Nanotechnol. Rev..

[9] Liang Z.W., Nemenyi A., Kovacs G.P., Gyuricza C. (2023). Potential use of bamboo resources in energy value-added conversion technology and energy systems. Glob. Change Biol. Bioenergy.

[10] Silva M.F., Menis-Henrique M.E.C., Felisberto M.H.F., Goldbeck R., Clerici M. (2020). Bamboo as an eco-friendly material for food and biotechnology industries. Curr. Opin. Food Sci..

[11] Cheng Y.Q., Wan S.Q., Yao L.N., Lin D., Wu T., Chen Y.J., Zhang A.L., Lu C.F. (2023). Bamboo leaf: A review of traditional medicinal property, phytochemistry, pharmacology, and purification technology. J. Ethnopharmacol..

[12] Hu R., Wu L., Liao X., Zhang F., Zheng J. (2023). Synergistic modification of ultrasound and bamboo leaf flavonoid on the rheological properties, multi-scale structure, and in vitro digestibility of pea starch. Food Chem..

[13] Chongtham N., Bisht M.S., Haorongbam S. (2011). Nutritional Properties of Bamboo Shoots:Potential and Prospects for Utilization as a Health Food. Compr. Rev. Food Sci. Food Saf..

[14] Srivastava R.C.J.C.S. (1990). Bamboo, new raw material for phytosterols. Curr. Sci..

[15] NSBC Database National Bureau of Statistics of China: 2020. http://www.stats.gov.cn/.

[16] Choudhury D., Sahu J.K., Sharma G.D. (2011). Bamboo shoot based fermented food products: A review. J. Sci. Ind. Res..

[17] Yang Q., Duan Z., Wang Z., He K., Sun Q., Peng Z. (2008). Bamboo resources, utilization and ex-situ conservation in Xishuangbanna, South-eastern China. J. For. Res..

[18] Tamang J.P. (2023). “Ethno-microbiology” of ethnic Indian fermented foods and alcoholic beverages. J. Appl. Microbiol..

[19] Chongtham N., Bisht M.S., Premlata T., Bajwa H.K., Sharma V., Santosh O. (2022). Quality improvement of bamboo shoots by removal of antinutrients using different processing techniques: A review. J. Food Sci. Technol. Mysore.

[20] Luo X., Wang Q., Zheng B., Lin L., Chen B., Zheng Y., Xiao J. (2017). Hydration properties and binding capacities of dietary fibers from bamboo shoot shell and its hypolipidemic effects in mice. Food Chem. Toxicol..

[21] Nirmala C., Bisht M.S., Laishram M. (2014). Bioactive compounds in bamboo shoots: Health benefits and prospects for developing functional foods. Int. J. Food Sci. Technol..

[22] Sarkar D., Chandra A.K., Chakraborty A., Ghosh S., Chattopadhyay S., Singh L.H., Ray I. (2020). Effects of bamboo shoots (Bambusa balcooa) on thyroid hormone synthesizing regulatory elements at cellular and molecular levels in thyrocytes. J. Ethnopharmacol..

[23] Chongtham N., Bisht M.S. (2020). Bamboo Shoot: Superfood for Nutrition, Health and Medicine.

[24] Devi Y.R., Chakma A., Yenkokpam S. (2017). Traditional wisdom of community regarding elimination of cyanogenic glycosides in bamboo shoot food products. Indian J. Tradit. Know.

[25] Panee J. (2015). Potential Medicinal Application and Toxicity Evaluation of Extracts from Bamboo Plants. J. Med. Plants Res..

[26] Chen G.J., Bu F., Chen X.H., Li C.F., Wang S.S., Kan J.Q. (2018). Ultrasonic extraction, structural characterization, physicochemical properties and antioxidant activities of polysaccharides from bamboo shoots (Chimonobambusa quadrangularis) processing by-products. Int. J. Biol. Macromol..

[27] Tang J.J., Zhang Z.X., Zheng S.L., Gao N., Li Z.J., Li K. (2021). Changes of Main Nutrient Components and Volatile Flavor Substances in Processing of Canned Bamboo Shoots. Fermentation.

[28] Satya S., Bal L.M., Singhal P., Naik S.N. (2010). Bamboo shoot processing: Food quality and safety aspect. Trends Food Sci. Technol..

[29] Chandramouli S., Viswanath S. (2012). Bamboo Shoots—An Emerging New Age Health Food. For. Bull..

[30] Sarangthem K., Singh T.N. (2003). Microbial bioconversion of metabolites from fermented succulent bamboo shoots into phytosterols. Curr. Sci. India.

[31] Wang X., Yang L., Geng X., Shi W., Chen Y., Lu C. (2023). Integrative analysis of metabolome and transcriptome reveals the different metabolite biosynthesis profiles related to palatability in winter and spring shoot in moso bamboo. Plant Physiol. Biochem. PPB.

[32] Liu X., Jiang P., Fu B., Yang J., Qi L., Shang S. (2023). Extraction and physiological functionality of dietary fiber from bamboo shoots and its application in food. Food Ferment. Ind..

[33] Ruihua B.A.I., Xingcui D., Shudong W. (2011). Nutritional and Safety Evaluation of Unearthed Bamboo Shoots during Growth. Food Sci..

[34] Zhen J., Zhan F.S., Zhou C.H., Kan J.Q. (2014). Comparison of Flavor Compounds in Fresh and Pickled Bamboo Shoots by GC-MS and GC-Olfactometry. Food Sci. Technol. Res..

[35] Lin Z., Chen J., Zhang J.Z., Brooks M.S.L. (2018). Potential for Value-Added Utilization of Bamboo Shoot Processing Waste-Recommendations for a Biorefinery Approach. Food Bioprocess. Technol..

[36] Fan L.L., Hu J.J., Guo Z.W., Chen S.L., He Q.J. (2023). Shoot Nutrition and Flavor Variation in Two Species: Does the Quality of Edible Bamboo Shoot Diaphragm and Flesh Differ?. Foods.

[37] Li M.M., Zhou Y., Zuo L., Nie D., Li X.A. (2021). Dietary fiber regulates intestinal flora and suppresses liver and systemic inflammation to alleviate liver fibrosis in mice. Nutrition.

[38] Choudhury D., Sahu J.K., Sharma G.D. (2012). Value addition to bamboo shoots: A review. J. Food Sci. Technol. Mysore.

[39] Xu Y., Ma C.-Y., Sun S.-C., Zhang C., Wen J.-L., Yuan T.-Q. (2023). Fractionation and evaluation of light-colored lignin extracted from bamboo shoot shells using hydrated deep eutectic solvents. Bioresour. Technol..

[40] Du J.J., Zhu Q., Guo J.G., Wu Y.H., Hu Z.Q., Yang S., Jiang J. (2023). Effects of ultrasonic and steam-cooking treatments on the physicochemical properties of bamboo shoots protein and the stability of O/W emulsion. Heliyon.

[41] Li J.W., Xi Y.H., Wu L.R., Zhang H. (2023). Preparation, characterization and digestion of bamboo shoot protein/soybean protein isolate based-oleogels by emulsion-templated approach. Food Hydrocolloid.

[42] Karanja P.N., Kenji G.M., Njoroge S.M., Sila D.N., Onyango C.A., Koaze H., Baba N. (2015). Variation of Nutrients and Functional Properties within Young Shoots of a Bamboo Species (*Yushania alpina*) Growing at Mt. Elgon Region in Western Kenya. J. Food Nutr. Res..

[43] Nirmala C., David E., Sharma M.L. (2007). Changes in nutrient components during ageing of emerging juvenile bamboo shoots. Int. J. Food Sci. Nutr..

[44] Lin Z., Jiao G.L., Zhang J.Z., Celli G.B., Brooks M.S.L. (2021). Optimization of protein extraction from bamboo shoots and processing wastes using deep eutectic solvents in a biorefinery approach. Biomass Convers. Bior.

[45] Nongdam P., Tikendra L. (2014). The Nutritional Facts of Bamboo Shoots and Their Usage as Important Traditional Foods of Northeast India. Int. Sch. Res. Not..

[46] Gao Q., Jiang H., Tang F., Cao H.Q., Wu X.W., Qi F.F., Sun J., Yang J. (2019). Evaluation of the bitter components of bamboo shoots using a metabolomics approach. Food Funct..

[47] Tanabe C., Furuta K., Maeda M., Kimura Y. (2017). Structural feature of N-glycans of bamboo shoot glycoproteins: Useful source of plant antigenic N-glycans. Biotechnol. Biochem..

[48] Edashige Y., Ishii T. (1998). Hemicellulosic polysaccharides from bamboo shoot cell-walls. Phytochemistry.

[49] Karanja P.N. (2017). Physicochemical Properties of Bamboo Shoots of Selected Species grown in Kenya and Utilization as Human Food. Ph.D. Dissertation.

[50] Deng J., Yun J., Gu Y., Yan B., Yin B., Huang C. (2023). Evaluating the In Vitro and In Vivo Prebiotic Effects of Different Xylo-Oligosaccharides Obtained from Bamboo Shoots by Hydrothermal Pretreatment Combined with Endo-Xylanase Hydrolysis. Int. J. Mol. Sci..

[51] Yang J.L., Wu L.R., Yang H.M., Pan Y.H. (2021). Using the Major Components (Cellulose, Hemicellulose, and Lignin) of Bamboo Shoot as Dietary Fiber. Front. Bioeng. Biotechnol..

[52] Chen G.J., Hong Q.Y., Ji N., Wu W.N., Ma L.Z. (2020). Influences of different drying methods on the structural characteristics and prebiotic activity of polysaccharides from bamboo shoot (*Chimonobambusa quadrangularis*) residues. Int. J. Biol. Macromol..

[53] Dai J., Xiao Z.Q., Li J.J., Ge Q., Wang H.P., Sha R.Y., Mao J.W. (2023). The structural characteristic of bamboo shoot shell polysaccharides extracted using ultrasound-assisted phosphotungstic acid hydrolysis and its protection against cell oxidative injury. Int. J. Food Sci. Technol..

[54] Chen C.H., Guan X.F., Liu X.Y., Zhuang W.J., Xiao Y.Q., Zheng Y.F., Wang Q. (2022). Polysaccharides from Bamboo Shoot (Leleba oldhami Nakal) Byproducts Alleviate Antibiotic-Associated Diarrhea in Mice through Their Interactions with Gut Microbiota. Foods.

[55] Wu W.J., Li Q., Chen H.J., Fang X.J., Niu B., Liu R.L., Mu H.L., Gao H.Y. (2023). In vitro fermentation characteristics of the dietary fiber in bamboo (*Phyllostachys edulis*) shoots and its regulatory effects on the intestinal microbiota and metabolites. Food Chem..

[56] Bajwa H.K., Santosh O., Koul A., Bisht M.S., Nirmala C. (2019). Quantitative determination of macroelement and microelement content of fresh and processed bamboo shoots by wavelength dispersive X-ray fluorescence spectrometry. X-Ray Spectrom..

[57] Saini N., Rawat K., Bisht M.S., Nirmala C., Saini N., Rawat K., Bisht M.S., Nirmala C. (2017). Qualitative and quantitative mineral element variances in shoots of two edible bamboo species after processing and storage evaluated by wavelength dispersion X-ray fluorescence spectrometry. Int. J. Innov. Res. Sci. Eng. Technol..

[58] Pandey A.K., Ojha V. (2014). Precooking processing of bamboo shoots for removal of anti-nutrients. J. Food Sci. Technol.-Mysore.

[59] Zheng J., Zhang F.S., Zhou C.H., Chen G.J., Lin M., Kan J.Q. (2013). Changes in amino acid contents, texture and microstructure of bamboo shoots during pickling process. Int. J. Food Sci. Technol..

[60] Zhang J.-J., Ji R., Hu Y.-Q., Chen J.-C., Ye X.-Q. (2011). Effect of three cooking methods on nutrient components and antioxidant capacities of bamboo shoot(Phyllostachys praecox C.D. Chu et C.S. Chao). J. Zhejiang Univ. Sci. B.

[61] Bhatt B.P., Singh K., Singh A. (2005). Nutritional values of some commercial edible bamboo species of the North Eastern Himalayan region, India. J. Bamboo Ratt..

[62] Satya S., Singhal P., Bal L.M., Sudhakar P. (2012). Bamboo shoot: A potential source of food security. Mediterr. J. Nutr. Metab..

[63] Feleke S. (2013). Site factor on nutritional content of Arundinaria alpina and Oxytenanthera abyssinica bamboo shoots in Ethiopia. J. Hortic. For..

[64] Chandramouli S., Viswanath S. (2015). Nutritional composition of edible bamboo shoots of some commercially important bamboo species in Peninsular India. J. Basic Life Sci..

[65] Santosh O., Bajwa H.K., Bisht M.S., Chongtham N. (2018). Freeze-dried bamboo shoot powder for food fortification: Enrichment of nutritional content and organoleptic qualities of fortified biscuits. MOJ Food Process. Technol..

[66] Sonar N.R., Vijayendra S.V.N., Prakash M., Saikia M., Halami P.M. (2015). Nutritional and functional profile of traditional fermented bamboo shoot based products from Arunachal Pradesh and Manipur states of India. Int. Food Res. J..

[67] Rawat K., Chongtham N., Bisht M.S. Processing Techniques for Reduction of Cyanogenic Glycosides from Bamboo Shoots. Proceedings of the 10th World Bamboo Congress.

[68] Law C., Exley C. (2011). New insight into silica deposition in horsetail (*Equisetum arvense*). Bmc Plant Biol..

[69] Park E.-J., Jhon D.-Y. (2013). The Nutritional Composition of Bamboo Shoots and the Effects of its Fiber on Intestinal Microorganisms. J. Korean Soc. Food Cult..

[70] Waikhom S.D., Louis B., Sharma C.K., Kumari P., Somkuwar B.G., Singh M.W., Talukdar N.C. (2013). Grappling the high altitude for safe edible bamboo shoots with rich nutritional attributes and escaping cyanogenic toxicity. Biomed. Res. Int..

[71] Christian A.L., Knott K.K., Vance C.K., Falcone J.F., Bauer L.L., Fahey G.C., Willard S., Kouba A.J. (2015). Nutrient and mineral composition during shoot growth in seven species of Phyllostachys and Pseudosasa bamboo consumed by giant panda. J. Anim. Physiol. Anim. Nutr..

[72] Sood S., Walia S., Gupta M., Sood A. (2013). Nutritional Characterization of Shoots and other Edible Products of an Edible Bamboo—Dendrocalamus hamiltonii. Curr. Res. Nutr. Food Sci. J..

[73] Ren Y., Ma Y.S., Zhang Z.D., Qiu L.Y., Zhai H.H., Gu R.M., Xie Y.P. (2019). Total Alkaloids from Bamboo Shoots and Bamboo Shoot Shells of Pleioblastus amarus (Keng) Keng f. and Their Anti-Inflammatory Activities. Molecules.

[74] Milani G., Curci F., Cavalluzzi M.M., Crupi P., Pisano I., Lentini G., Clodoveo M.L., Franchini C., Corbo F. (2020). Optimization of Microwave-Assisted Extraction of Antioxidants from Bamboo Shoots of *Phyllostachys pubescens*. Molecules.

[75] Ogbe R.J., Ochalefu D.O., Mafulul S.G., Olaniru O. (2015). A review on dietary phytosterols: Their occurrence, metabolism and health benefits. Asian J. Plant Sci. Res..

[76] Lu B., Bao J., Shan L., Zhang Y. (2009). Technology for supercritical CO2 extraction of bamboo shoot oil and components of product. Trans. Chin. Soc. Agric. Eng..

[77] Xi Y.H., Zhang A.P., Wang Z.J., Farooq S., Zhang C., Wu L.R., Zhang H. (2021). Improved oxidation stability of camellia oil-in-water emulsions stabilized by the mixed monolayer of soy protein isolate/bamboo shoot protein complexes. Front. Nutr..

[78] Su Y., Dong H.L., Li M., Lai C.H., Huang C.X., Yong Q. (2019). Isolation of the Flavonoid from Bamboo Residues and Its Application as Metal Ion Sensor in Vitro. Polymers.

[79] Pandey A.K., Ojha V. (2012). Standardization of harvesting age of bamboo shoots with respect to nutritional and anti-nutritional components. J. For. Res..

[80] Sansenya S., Payaka A., Mansalai P. (2023). Biological activity and inhibition potential against ?-glucosidase and ?-amylase of 2,4-di-tert-butylphenol from bamboo shoot extract by in vitro and in silico studies. Process Biochem..

[81] Zulkafli Z.D., Wang H., Miyashita F., Utsumi N., Tamura K. (2014). Cosolvent-modified supercritical carbon dioxide extraction of phenolic compounds from bamboo leaves (*Sasa palmata*). J. Supercrit. Fluid..

[82] Chen F.P., Liu L.L., Tang C.H. (2020). Spray-drying microencapsulation of curcumin nanocomplexes with soy protein isolate: Encapsulation, water dispersion, bioaccessibility and bioactivities of curcumin. Food Hydrocolloid.

[83] Li J.Q., Li W.T., Deng Z.Y., Li H.Y., Yu Y., Zhang B. (2021). Comparison of free, conjugated, and insoluble-bound phenolics and their antioxidant activities in oven-drying and freeze-drying bamboo (*Phyllostachys edulis*) shoot tips. J. Food Sci..

[84] Xiao J.P., Li A.P., Tang Y.M., Li D.Y., Yang P., Cheng H.X.Z. (2022). Bound phenolics release from dried bamboo shoots prepared by different processes during gastrointestinal digestion: Bioaccessibility and bioactivity. Int. J. Food Sci. Technol..

[85] Sharma V. (2018). Changes in Bioactive Components and Anti-Nutrients During Processing of Bamboo Shoots.

[86] Yi J., Li M., Yang M., Cai S., Zhao T., Cao J., Cheng G. (2021). Characterisation and in vitro cytotoxicity of toxic and degradation compounds in bamboo shoots (*Dendrocalamus Sinicus*) during traditional fermentation. Int. J. Food Sci. Technol..

[87] Ding M., Wang K. (2018). Determination of cyanide in bamboo shoots by microdiffusion combined with ion chromatography–pulsed amperometric detection. R. Soc. Open Sci..

[88] Devi T. (2019). Edible bamboos of Manipur: Analysis of Nutrients, Antinutrients and Bioactive Compounds in Young Shoots. Ph.D. Thesis.

[89] Da J. (1998). Why are so many food plants cyanogenic?. Phytochemistry.

[90] Satya S.S.P., Prabhu V.G., Bal L.M., Sudhakar P. Exploring the nutraceutical potential and food safety aspect of bamboo shoot of some Indian species. Proceedings of the VIII World Bamboo Conference.

[91] Pokhariya P., Tangariya P., Sahoo A., Awasthi P., Pandey A. (2018). Reducing hydrocyanic acid content, nutritional and sensory quality evaluation of edible bamboo shoot based food products. Int. J. Chem. Sci..

[92] Fekadu Gemede H. (2014). Antinutritional Factors in Plant Foods: Potential Health Benefits and Adverse Effects. Int. J. Nutr. Food Sci..

[93] Oleszek M., Oleszek W. (2020). Saponins in Food. Handbook of Dietary Phytochemicals.

[94] Kim H.J., Kim B., Lee M.R., Ra M., Lee Y. (2022). Bamboo Shoot and Artemisia capillaris Extract Mixture Ameliorates Dextran Sodium Sulfate-Induced Colitis. Curr. Issues Mol. Biol..

[95] Gao Y., Zong Z.H., Xia W., Fang X.J., Liu R.L., Wu W.J., Mu H.L., Han Y.C., Xiao S.Y., Gao H.Y. (2023). Hepatoprotective effect of water bamboo shoot (*Zizania latifolia*) extracts against acute alcoholic liver injury in a mice model and screening of bioactive phytochemicals. Food Front..

[96] Zhou X.L., Pak S., Li D.T., Dong L., Chen F., Hu X.S., Ma L.J. (2023). Bamboo Shoots Modulate Gut Microbiota, Eliminate Obesity in High-Fat-Diet-Fed Mice and Improve Lipid Metabolism. Foods.

[97] Li D., Limwachiranon J., Li L., Zhang L., Xu Y., Fu M., Luo Z. (2019). Hydrogen peroxide accelerated the lignification process of bamboo shoots by activating the phenylpropanoid pathway and programmed cell death in postharvest storage. Postharvest Biol. Technol..

[98] Pramod Kumar G.N., Chandrakant H.V., Verghese A.J., Manjunatha B., Balaraj B.M., Sudharshan Murthy K.A. (2011). Bamboo Shoot—Is it Edible or Poisonous?. J. Indian Soc. Toxicol..

[99] Ahmed A., Zulkifli I., Farjam A.S., Abdullah N., Liang J.B., Awad E.A. (2014). Effect of solid state fermentation on nutrient content and ileal amino acids digestibility of canola meal in broiler chickens. Ital. J. Anim. Sci..

[100] Hassan G., Yusuf L., Adebolu T., Onifade A. (2015). Effect of fermentation on mineral and anti-nutritional composition of cocoyam (Colocasia esculenta linn). Sky J. Food Sci..

[101] Kong C.K., Tan Y.N., Chye F.Y., Sit N.W. (2020). Nutritional composition and biological activities of the edible shoots of Bambusa vulgaris and Gigantochloa ligulata. Food Biosci..

[102] Sarangthem K., Singh T.N. (2013). Fermentation decreases the antinutritional content in bamboo shoots. Int. J. Curr. Microbiol. Appl. Sci..

[103] Li C.T., Xuan L.L., He Y.M., Wang J., Zhang H., Ying Y.Q., Wu A.M., Bacic A., Zeng W., Song L.L. (2019). Molecular Mechanism of Xylogenesis in Moso Bamboo (*Phyllostachys edulis*) Shoots during Cold Storage. Polymers.

[104] Luo Z.S., Xu X.L., Yan B.F. (2008). Use of 1-methylcyclopropene for alleviating chilling injury and lignification of bamboo shoot (*Phyllostachys praecox f. prevernalis*) during cold storage. J. Sci. Food Agric..

[105] Gorni C., Allemand D., Rossi D., Mariani P. (2015). Microbiome profiling in fresh-cut products. Trends Food Sci. Technol..

[106] Wang D., Li L., Xu Y.Q., Limwachiranon J., Li D., Ban Z.J., Luo Z.S. (2017). Effect of Exogenous Nitro Oxide on Chilling Tolerance, Polyamine, Proline, and gamma-Aminobutyric Acid in Bamboo Shoots (Phyllostachys praecox f. prevernalis). J. Agric. Food Chem..

[107] Badwaik L.S., Borah P.K., Deka S.C. (2014). Antimicrobial and enzymatic antibrowning film used as coating for bamboo shoot quality improvement. Carbohyd Polym..

[108] Luo Z.S., Xu X.L., Yan B.F. (2008). Accumulation of lignin and involvement of enzymes in bamboo shoot during storage. Eur. Food Res. Technol..

[109] Li X.Y., Xie L.H., Zheng H.F., Cai M.M., Cheng Z.C., Bai Y.C., Li J., Gao J. (2019). Transcriptome profiling of postharvest shoots identifies—and -promoted shoot senescence. Tree Physiol..

[110] Yu L.X., Pei J.L., Zhao Y.H., Wang S.G. (2021). Physiological Changes of Bamboo (*Fargesia yunnanensis*) Shoots During Storage and the Related Cold Storage Mechanisms. Front. Plant Sci..

[111] Xu J., Ji N., Wang R., Ma C., Lei J., Zhang N., Liu R., Deng Y. (2023). Study on the Regulation Mechanism of 1-MCP Combined with SO2 Treatment on Postharvest Senescence of Bamboo Shoots (*Chimonobambusa quadrangularis*) in Karst Mountain Area. Agronomy.

[112] Zheng X., Gong M., Zhang Q., Tan H., Li L., Tang Y., Li Z., Peng M., Deng W. (2022). Metabolism and Regulation of Ascorbic Acid in Fruits. Plants.

[113] Bhowmik P.K., Matsui T. (2005). Ethylene biosynthetic genes in ‘Moso’ bamboo shoot in response to wounding. Postharvest Biol. Technol..

[114] Zheng J., Li S.E., Xu Y.H., Zheng X.L. (2019). Effect of oxalic acid on edible quality of bamboo shoots (Phyllostachys prominens) without sheaths during cold storage. LWT-Food Sci. Technol..

[115] Li C.T., Suo J.W., Xuan L.L., Ding M.Z., Zhang H., Song L.L., Ying Y.Q. (2019). Bamboo shoot-lignification delay by melatonin during low temperature storage. Postharvest Biol. Tec..

[116] Zhang H., Ying Y.Q., Wang J., Zhao X.H., Zeng W., Beahan C., He J.B., Chen X.Y., Bacic A., Song L.L. (2018). Transcriptome analysis provides insights into xylogenesis formation in Moso bamboo (*Phyllostachys edulis*) shoot. Sci. Rep..

[117] Zhang Z.Y., Li C.T., Zhang H., Ying Y.Q., Hu Y.Y., Song L.L. (2020). Comparative Analysis of the Lignification Process of Two Bamboo Shoots Stored at Room Temperature. Plants.

[118] Zeng F.F., Jiang T.J., Wang Y.S., Luo Z.S. (2015). Effect of UV-C treatment on modulating antioxidative system and proline metabolism of bamboo shoots subjected to chilling stress. Acta Physiol. Plant.

[119] Huang Y.J., Xun H., Yi G.L., Li T., Yao X., Tang F. (2022). Integrated Metabolomic and Transcriptomic Analysis Reveals the Effect of Artificial Shading on Reducing the Bitter Taste of Bamboo Shoots. Horticulturae.

[120] Holdsworth S.D. (2017). Fruit and Vegetable Preservation. The Preservation of Fruit and Vegetable Food Products.

[121] Yang H.Q., Zhou C.S., Wu F.H., Cheng J.Y. (2010). Effect of nitric oxide on browning and lignification of peeled bamboo shoots. Postharvest Biol. Tec..

[122] Song L.L., Gao H.Y., Chen W.X., Chen H.J., Mao J.L., Zhou Y.J., Duan X.W., Joyce D.C. (2011). The role of 1-methylcyclopropene in lignification and expansin gene expression in peeled water bamboo shoot (*Zizania caduciflora* L.). J. Sci. Food Agric..

[123] Yang H.Q., Zheng J.Y., Huang C.Q., Zhao X.F., Chen H.Y., Sun Z.D. (2015). Effects of Combined Aqueous Chlorine Dioxide and Chitosan Coatings on Microbial Growth and Quality Maintenance of Fresh-Cut Bamboo Shoots (Phyllostachys praecox f. prevernalis.) During Storage. Food Bioprocess. Technol..

[124] Wang J.X., Jiang J., Wang J., Wang Z.X., Yang X.P., Jia L.R. (2019). The influence of gamma irradiation on the storage quality of bamboo shoots. Radiat. Phys. Chem..

[125] Liu D.J., Wang F., Brennan C., Benjakul S., Xiao G.S., Ying X.G., Ma L.K. (2023). Combined melatonin and UV-C treatment maintains the quality of fresh-cut bamboo shoots during storage by altering microbial diversity and metabolites. Postharvest Biol. Technol..

[126] Chen H.Y., Ling J.G., Wu F.H., Zhang L.J., Sun Z.D., Yang H.Q. (2013). Effect of hypobaric storage on flesh lignification, active oxygen metabolism and related enzyme activities in bamboo shoots. LWT-Food Sci. Technol..

[127] Luo Z.S., Wu X., Xie Y., Chen C. (2012). Alleviation of chilling injury and browning of postharvest bamboo shoot by salicylic acid treatment. Food Chem..

[128] Liu R., Wang H., Yang H., Zhang H., Chen J., Gao H., Chen H. (2022). Effect of ozone treatment on lignification and postharvest quality of water bamboo shoots. eFood.

[129] Chen L., Huang J., Yang R., Zhou Z., Liu X., Bi X. (2021). Preservation Effect of Ozone Treatment Combined with Modified Atmosphere Packaging on Moso Bamboo Shoots. Food Sci..

[130] Zeng F.F., Luo Z.S., Xie J.W., Feng S.M. (2015). Gamma radiation control quality and lignification of bamboo shoots (*Phyllostachys praecox* f. *prevemalis.*) stored at low temperature. Postharvest Biol. Tec..

[131] Song L.L., Chen H.J., Gao H.Y., Fang X.J., Mu H.L., Yuan Y., Yang Q., Jiang Y.M. (2013). Combined modified atmosphere packaging and low temperature storage delay lignification and improve the defense response of minimally processed water bamboo shoot. Chem. Cent. J..

[132] Bal L.M., Singhal P., Satya S., Naik S.N., Kar A. (2012). Bamboo Shoot Preservation for Enhancing its Business Potential and Local Economy: A Review. Crit. Rev. Food Sci..

[133] Wen B., Cheng Z., Hu Y., Boon-Ek Y., Wongs-Aree C., Supapanich S.J.P.B. (2019). Ultraviolet-C treatment maintains physicochemical quality of water bamboo (*Zizania latifolia*) shoots during postharvest storage. Postharvest Biol. Technol..

[134] Miao M., Wang Q., Zhang T., Jiang B. (2011). Effect of high hydrostatic pressure (HHP) treatment on texture changes of water bamboo shoots cultivated in China. Postharvest Biol. Technol..

[135] Cuadrado C., Allaf K. (2007). Molecules. Special Issue: Opportunities and Challenges in High Pressure Processing of Foods Special Issue. Food Sci. Nutr..

[136] Li X., Xing Y., Shui Y., Cao X., Xu R., Xu Q., Bi X., Liu X. (2021). Quality of bamboo shoots during storage as affected by high hydrostatic pressure processing. Int. J. Food Prop..

[137] Liu Z.L., Li L., Luo Z.S., Zeng F.F., Jiang L., Tang K.C. (2016). Effect of brassinolide on energy status and proline metabolism in postharvest bamboo shoot during chilling stress. Postharvest Biol. Technol..

[138] Yang H.Q., Wu F.H., Cheng J.Y. (2011). Effects of nitric oxide treatment on active oxygen metabolism and flesh lignification in bamboo shoots. J. Hortic. Sci. Biotechnol..

[139] Zheng J., Li S.E., Ali M., Huang Q.H., Zhneg X.L., Pang L.J. (2020). Effects of UV-B treatment on controlling lignification and quality of bamboo (*Phyllostachys prominens*) shoots without sheaths during cold storage. J. Integr. Agric..

[140] Shen Q., Kong F., Wang Q. (2006). Effect of modified atmosphere packaging on the browning and lignification of bamboo shoots. J. Food Eng..

[141] Li S.B., Tian Y.F., Sun M.H., Liu J.J., Bai Y.X., Liu X.L., Guo Y. (2022). Characterization of Key Aroma Compounds in Fermented Bamboo Shoots Using Gas Chromatography-Olfactometry-Mass Spectrometry, Odor Activity Values, and Aroma Recombination Experiments. Foods.

[142] Behera P., Balaji S. (2021). Health Benefits of Fermented Bamboo Shoots: The Twenty-First Century Green Gold of Northeast India. Appl. Biochem. Biotechnol..

[143] Bajwa H.K., Santosh O., Koul A., Bisht M.S., Nirmala C. (2019). Phytomodulatory effects of fresh and processed shoots of an edible bamboo Dendrocalamus hamiltonii Nees & Arn. Ex Munro on antioxidant defense system in mouse liver. J. Food Meas. Charact..

[144] Yuan L., Lu L.X., Koutsimanis G., Ge C.F., Johnson D.P. (2019). Research on the high hydrostatic pressure and microwave combined inactivation process and the application to boiled bamboo shoots. J. Food Saf..

[145] Gao Q., Wang D., Shao S., Xue Y., Zhang Y., Chen C., Tang F., Sun J., Li Y., Guo Q. (2020). Identification and quantitation of the actual active components in bamboo juice and its oral liquid by NMR and UPLC-Q-TOF-MS. Sci. Rep..

[146] Dadwal V., Sharma A., Joshi R., Gupta M.J.F.S. (2022). Assessment of nutritional properties and phenolic characterization of freshly harvestedDendrocalamus hamiltonishoots and processed bamboo candy. Food Sci. Biotechnol..

[147] Zhao C.M., Du T., Li P., Du X.J., Wang S. (2021). Production and Characterization of a Novel Low-Sugar Beverage from Red Jujube Fruits and Bamboo Shoots Fermented with Selected *Lactiplantibacillus plantarum*. Foods.

[148] Pattarathitiwat P., Chinvongamorn C., Sansenya S. (2021). Evaluation of Cyanide Content, Volatile Compounds Profile, and Biological Properties of Fresh and Boiled Sliced Thai Bamboo Shoot (*Dendrocalamus asper* Back.). Prev. Nutr. Food Sci..

[149] Xu X.Y., Xie W.G., Xiang C., You Q., Tian X.G. (2023). Predicting the dietary fiber content of fresh-cut bamboo shoots using a visible and near-infrared hyperspectral technique. J. Food Meas. Charact..

[150] Li J.J., Liu Y., Xiao H., Huang H., Deng G.W., Chen M.J., Jiang L.W. (2022). Bacterial communities and volatile organic compounds in traditional fermented salt-free bamboo shoots. Food Biosci..

[151] Singhal P., Satya S., Naik S.N. (2021). Fermented bamboo shoots: A complete nutritional, anti-nutritional and antioxidant profile of the sustainable and functional food to food security. Food Chem.-Mol. Sci..

[152] Chi H., Lu W.W., Liu G.Q., Qin Y.Y. (2020). Physiochemical property changes and mineral element migration behavior of bamboo shoots during traditional fermentation process. J. Food Process Pres..

[153] Zhang Y., Wu L., Li Y., Yang J., Yang H., Zhao Y., Chen G. (2024). Bamboo shoot and its food applications in last decade: An undervalued edible resource from forest to feed future people. Trends Food Sci. Technol..

[154] Thounaojam P., Bisht M., Nirmala C. (2017). Effect of processing on nutritional and phytochemical contents in shoots of an edible bamboo Dendrocalamus latiflorus Munro. Int. J. Agric. Sci..

[155] Fu S.-G., Yoon Y., Bazemore R. (2002). Aroma-active components in fermented bamboo shoots. J. Agric. Food Chem..

[156] Zhang F.S., Zheng J., Zhong J.F., Chen G.J., Kan J.Q. (2017). Kinetics of Texture Change of Bamboo Shoots During Pickling Process. J. Food Process Eng..

[157] Badwaik L.S., Choudhury S., Borah P.K., Sit N., Deka S.C. (2014). Comparison of Kinetics and Other Related Properties of Bamboo Shoot Drying Pretreated with Osmotic Dehydration. J. Food Process. Preserv..

[158] Singhal P., Rudra S.G., Singh R.K., Satya S., Naik S.N. (2018). Impact of drying techniques on physical quality of bamboo shoots: Implications on tribal’s livelihoods. Indian J. Tradit. Knowl..

[159] Bal L.M., Yogranjan, Naik S.N., Satya S., Kar A. (2017). Changes in tissue structure and physico-chemical quality characteristics of bamboo shoot slices during microwave drying process. J. Food Meas. Charact..

[160] Chen X.G., Zhu B., Zhan-Rong H.E. (2012). Research Progress of the Use of Bamboo Shoots Processing Tail Materials. Food Nutr. China.

[161] Devi O.J., Pamba P. (2015). Antihypertensive activity of bamboo shoot: A review. Asian J. Pharm. Clin. Res..

[162] Chen G.J., Chen X.H., Yang B., Yu Q.Q., Wei X.Y., Ding Y.B., Kan J.Q. (2019). New insight into bamboo shoot (*Chimonobambusa quadrangularis*) polysaccharides: Impact of extraction processes on its prebiotic activity. Food Hydrocolloid.

[163] Gilbert-López B., Barranco A., Herrero M., Cifuentes A., Ibáñez E. (2017). Development of new green processes for the recovery of bioactives from Phaeodactylum tricornutum. Food Res. Int..

[164] Duan L., Dou L.L., Guo L., Li P., Liu E.H. (2016). Comprehensive Evaluation of Deep Eutectic Solvents in Extraction of Bioactive Natural Products. ACS Sustain. Chem. Eng..

[165] Du J.J., Yang S., Zhu Q., Wu Y.H., Guo J.G., Jiang J. (2021). Preparation and characterization of thermoplastic starch/bamboo shoot processing by-product microcrystalline cellulose composites. Biomass Convers. Biorefinery.

[166] Contreras M.d.M., Lama-Muñoz A., Gutiérrez-Pérez J.M., Espínola F., Moya M., Castro E. (2019). Protein extraction from agri-food residues for integration in biorefinery: Potential techniques and current status. Bioresour. Technol..

[167] Yang M., Wu L.R., Cao C.J., Wang S.Y., Zhang D.M. (2021). Extrusion improved the physical and chemical properties of dietary fibre from bamboo shoot by-products. Int. J. Food Sci. Technol..

[168] Fang D.Y., Wang Q., Chen C.H., Li Z.Y., Li S.T., Chen W., Zheng Y.F. (2021). Structural characteristics, physicochemical properties and prebiotic potential of modified dietary fibre from the basal part of bamboo shoot. Int. J. Food Sci. Technol..

[169] Wang Y.B., Zhang Y.L., Cheng J.W., Zhao J.C., Shi R., He L., Li Q., Chen Y.J. (2022). Efficient purification of flavonoids from bamboo shoot residues of by macroporous resin and their hypoglycemic activity. Food Chem. X.

[170] Fang Y., Si B., Qiu J., Wen Q., An M., Wang B., Jiang W. (2020). Bioconversion of bamboo shoot shell to methane assisted by microwave irradiation and fungus metabolism. Sci. Total Environ..

[171] Yang Y.Z., Fan F.W., Xie J.P., Fang K.Y., Zhang Q., Chen Y.R., Cao X.W., Deng Z.M. (2023). Isolation and characterization of cellulosic fibers from bamboo shoot shell. Polym. Bull..

[172] Gao Q., Ni L.M., He Y.Y., Hou Y.M., Hu W.H., Liu Z.J. (2022). Effect of hydrothermal pretreatment on deashing and pyrolysis characteristics of bamboo shoot shells. Energy.

[173] Lin T., Wang Q., Zheng X., Chang Y., Cao H., Zheng Y.F. (2022). Investigation of the Structural, Thermal, and Physicochemical Properties of Nanocelluloses Extracted From Bamboo Shoot Processing Byproducts. Front. Chem..

[174] Tang W., Tang Z.Y., Qian H.J., Huang C.X., He Y.C. (2023). Implementing dilute acid pretreatment coupled with solid acid catalysis and enzymatic hydrolysis to improve bioconversion of bamboo shoot shells. Bioresour. Technol..

[175] Peng X.W., Liu J.J., Tang N., Deng J., Liu C., Kan H., Zhao P., Zhang X., Shi Z.J., Liu Y. (2023). Sequential extraction, structural characterization, and antioxidant activity of polysaccharides from Dendrocalamus brandisii bamboo shoot shell. Food Chem. X.

[176] Xu Y., Liu Y.H., Xu L.H., He Y.T., Wen J.L., Yuan T.Q. (2023). Enhancing saccharification of bamboo shoot shells by rapid one-pot pretreatment of hydrated deep eutectic solvent. Bioresour. Technol..

[177] Li H.B., He Z.X., Jiang Y.Z., Kan J., Peng T., Zhong M.Q., Hu Z. (2021). Bioconversion of bamboo shoot shells through the cultivation of the edible mushroomsVolvariella volvacea. Ecotoxicology.

[178] Liu L.L., Liu L.Y., Lu B.Y., Chen M.Q., Zhang Y. (2013). Evaluation of Bamboo Shoot Peptide Preparation with Angiotensin Converting Enzyme Inhibitory and Antioxidant Abilities from Byproducts of Canned Bamboo Shoots. J. Agric. Food Chem..

[179] Huang Y.H., Peng Y.J., Yang Z., Chen S.Y., Liu J., Wang Z., Wang G., Lan S.L. (2022). Effects of Fermented Bamboo Shoot Processing Waste on Growth Performance, Serum Parameters, and Gut Microbiota of Weaned Piglets. Animals.

[180] Sarkar D., Chandra A.K., Chattopadyay S., Biswas M., Das S., Singh L.H., Ray I. (2021). Possible mechanism of bamboo shoots (*Bambusa balcooa*) induced thyroid disruption—Anin vitrostudy. Hum. Exp. Toxicol..

[181] Sawarkar A.D., Shrimankar D.D., Kumar M., Kumar P., Singh L. (2023). Bamboos as a cultivated medicinal grass for industries: A systematic review. Ind. Crops Prod..

